# Mild expression differences of *MECP2* influencing aggressive social behavior

**DOI:** 10.1002/emmm.201303744

**Published:** 2014-03-20

**Authors:** Martesa Tantra, Christian Hammer, Anne Kästner, Liane Dahm, Martin Begemann, Chiranjeevi Bodda, Kurt Hammerschmidt, Ina Giegling, Beata Stepniak, Aracely Castillo Venzor, Bettina Konte, Begun Erbaba, Annette Hartmann, Asieh Tarami, Walter Schulz-Schaeffer, Dan Rujescu, Ashraf U Mannan, Hannelore Ehrenreich

**Affiliations:** 1 Clinical Neuroscience, Max Planck Institute of Experimental MedicineGöttingen, Germany; 2 DFG Center for Nanoscale Microscopy & Molecular Physiology of the Brain (CNMPB)Göttingen, Germany; 3 Institute of Human Genetics, University Medical Center GöttingenGöttingen, Germany; 4 Cognitive Ethology Laboratory, German Primate CenterHalle, Germany; 5 Department of Psychiatry, University of HalleHalle, Germany; 6 Department of Neuropathology, University Medical Center GöttingenGöttingen, Germany

**Keywords:** genetic background, human, microRNA, mouse, phenotype-based genetic association study

## Abstract

The X-chromosomal *MECP2/Mecp2* gene encodes methyl-CpG-binding protein 2, a transcriptional activator and repressor regulating many other genes. We discovered in male FVB/N mice that mild (∼50%) transgenic overexpression of *Mecp2* enhances aggression. Surprisingly, when the same transgene was expressed in C57BL/6N mice, transgenics showed reduced aggression and social interaction. This suggests that *Mecp2* modulates aggressive social behavior. To test this hypothesis in humans, we performed a phenotype-based genetic association study (PGAS) in >1000 schizophrenic individuals. We found *MECP2* SNPs rs2239464 (G/A) and rs2734647 (C/T; 3′UTR) associated with aggression, with the G and C carriers, respectively, being *more* aggressive. This finding was replicated in an independent schizophrenia cohort. Allele-specific *MECP2*mRNA expression differs in peripheral blood mononuclear cells by ∼50% (rs2734647: C > T). Notably, the brain-expressed, species-conserved miR-511 binds to *MECP2* 3′UTR only in T carriers, thereby suppressing gene expression. To conclude, subtle *MECP2/Mecp2* expression alterations impact aggression. While the mouse data provides evidence of an interaction between genetic background and mild *Mecp2 over*expression, the human data convey means by which genetic variation affects *MECP2* expression and behavior.

## Introduction

The X-chromosomal *MECP2/Mecp2* gene encodes for methyl-CpG-binding protein 2, which can act both as a transcriptional activator and repressor (Chao & Zoghbi, 2012). Indeed, hundreds of genes have been estimated to be regulated, directly or indirectly, by this protein (Chahrour *et al*, 2008; Cohen *et al*, 2008). Complete or partial *loss-of-function* mutations of *MECP2* lead to Rett syndrome, characterized by a gender-dependent array of symptoms, ranging from early loss of acquired speech and motor skills to severe mental retardation and neonatal encephalopathy, among many others (Amir *et al*, 1999; Bienvenu & Chelly, 2006). Interestingly, gene duplication can cause very similar symptoms (Ramocki *et al*, 2010), and both down- and upregulation of *MECP2* are associated with behavioral core features of autism spectrum disorders (ASD) (Peters *et al*, 2013).

Diverse genetic mouse models, ranging from complete loss-of-function to reduced or enhanced expression, have been generated to study consequences of *Mecp2* mutations (Chen *et al*, 2001; Guy *et al*, 2001; Shahbazian *et al*, 2002; Collins *et al*, 2004; Moretti *et al*, 2005; Samaco *et al*, 2008; Bodda *et al*, 2013). In fact, there is a correlation between *Mecp2* “dosage” and phenotype severity, indicating a narrow normal expression range, with deviance in both directions being disadvantageous (Chao & Zoghbi, 2012). Along these lines, we have previously shown that mild overexpression of *Mecp2* of ∼1.4–1.5 times the wildtype (WT) level induces increased seizure propensity and aggression in male FVB/N mice, together with alterations in neuronal branching sites and augmented spine density (Bodda *et al*, 2013). Application of an epileptogenic compound, pentylenetetrazole, to transgenic (TG) neurons *in vitro* leads to a marked increase in amplitude and frequency of calcium spikes (Bodda *et al*, 2013).

While epileptic seizure susceptibility is a well-established result of alterations in *MECP2* expression (Shahbazian *et al*, 2002; Collins *et al*, 2004; Chahrour & Zoghbi, 2007; Chao *et al*, 2010; Chao & Zoghbi, 2012; Bodda *et al*, 2013), information on the behavioral consequences of mild overexpression is still rather limited. Doubling the *Mecp2* expression level in mice resulted in impaired social interaction (Samaco *et al*, 2012). *Mecp2* deficiency, however, also led to abnormal social behavior such as deficits in nest building, altered social interaction and enhanced aggression (Shahbazian *et al*, 2002; Moretti *et al*, 2005; Fyffe *et al*, 2008; Kerr *et al*, 2008; Samaco *et al*, 2008, 2009; Chao *et al*, 2010; Pearson *et al*, 2012). Taken together, tightly regulated *Mecp2* expression is obviously critical for normal function of genes involved in social behavior (Moretti *et al*, 2005).

Importantly, the reported phenotypes arose from diverse genetic backgrounds of mice carrying *Mecp2* mutations, not considering basic phenotypical differences among mouse strains (Wolfer & Lipp, 2000; Moy *et al*, 2009; Pietropaolo *et al*, 2011; O'Leary *et al*, 2013; Samaco *et al*, 2013). For example, behavioral comparisons between FVB/N and C57BL/6N strains revealed that FVB/N mice showed a higher frequency of bouts during behavioral paradigms of aggression (Mineur & Crusio, 2002; Pugh *et al*, 2004). Thus, the genetic background might well mask or modulate phenotypical changes induced by alterations in *Mecp2* expression.

In humans, data on aggression/impulsivity in Rett or *MECP2* gene duplication syndrome are scarce. A family study characterizing neuropsychiatric phenotypes of 9 males and 9 females with *MECP2* duplications revealed a high prevalence of hostility (63%) in female carriers. Moreover, an asymptomatic Rett mutation carrier with the mutation located in the deletion hotspot of the *MECP2* 3′ region has been reported to experience episodes of uncontrolled aggression (Huppke *et al*, 2006).

This study has been designed (1) to explore the role of gender and genetic background (FVB/N versus C57BL/6N) for behavioral phenotypes associated with mild *Mecp2* overexpression in mice, that is, altered aggression, spontaneous home cage behavior and predisposition to epileptic seizures, and (2) in parallel to search in humans for social behavioral consequences of common single-nucleotide polymorphisms (SNPs) within *MECP2* and (3) to strive for mechanistic insight into SNP-related *MECP2* expression differences in man.

## Results

### Mild *Mecp2* overexpression in FVB/N and C57BL/6N mice of both genders leaves most basic behaviors unaltered but modulates spontaneous home cage activity

To estimate the expression of the TG protein Mecp2^WT_EGFP^ (∼100 kDa) compared to endogenous Mecp2 (∼70 kDa) in TG mice, we performed Western blot analysis with total protein extracted from hippocampus and cerebellum. As described previously for FVB/N mice (Bodda *et al*, 2013), the relative expression of Mecp2^WT_EGFP^ in brain amounts to 40–50% of endogenous Mecp2 in both strains, leading to a subtle overexpression of ∼1.4- to 1.5-fold compared to WT. *Mecp2*^*WT_EGFP*^ TG mice of both genders and strains (FVB/N and C57BL/6N) develop and reproduce normally, and are devoid of any immediately obvious phenotype. Also, upon comprehensive testing of several independent cohorts of both genotypes and genders at different ages, all major domains of basic behavior, that is, general activity, anxiety, motor functions, exploratory behavior, sensory and cognitive functions were unaltered when compared to the respective WT littermate controls (Table 1a,b), thus confirming and extending our earlier report on male FVB/N TG mice (Bodda *et al*, 2013).

**Table 1:** (A) FVB/N mice

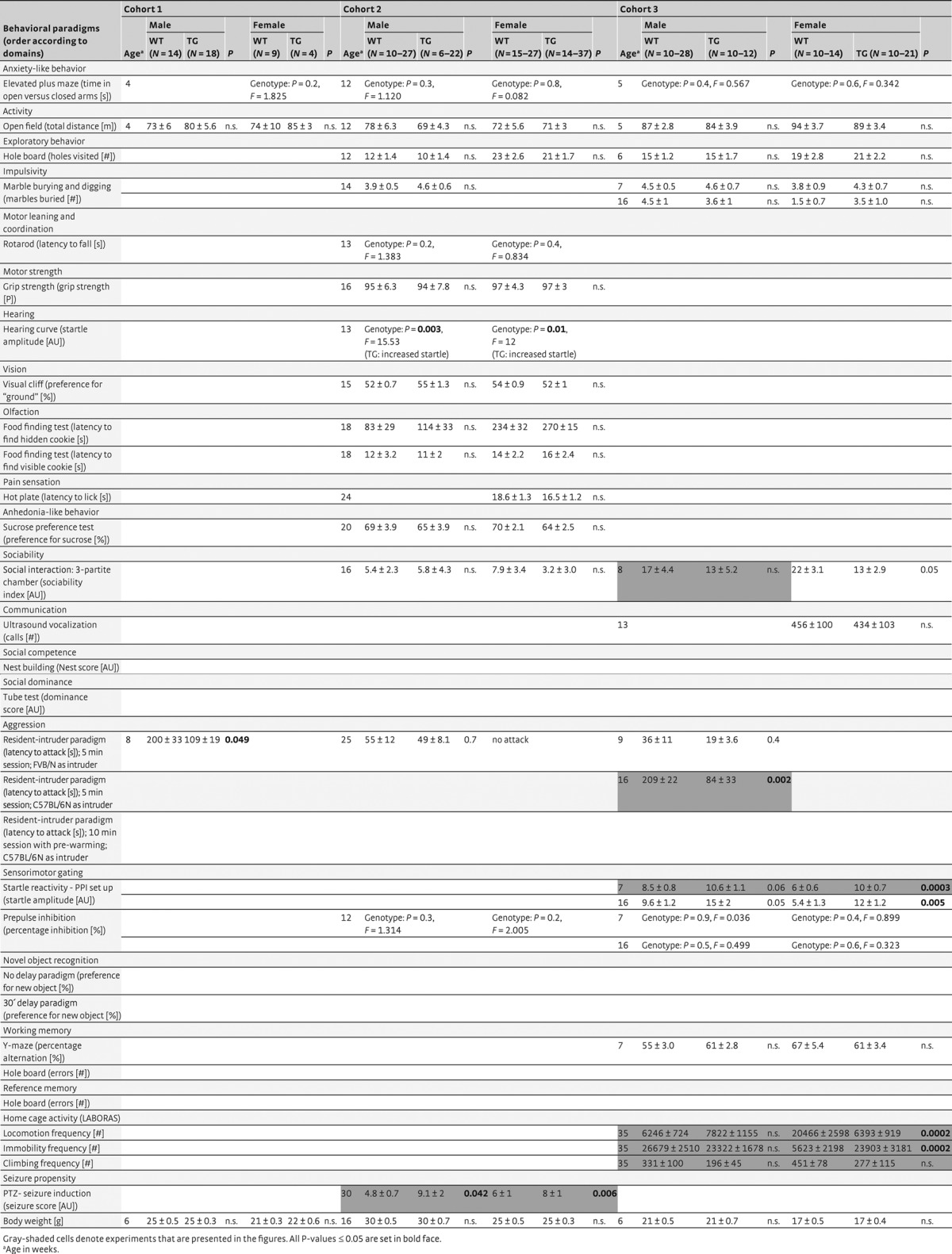

**Table 1:** (B) C57BL/6N mice

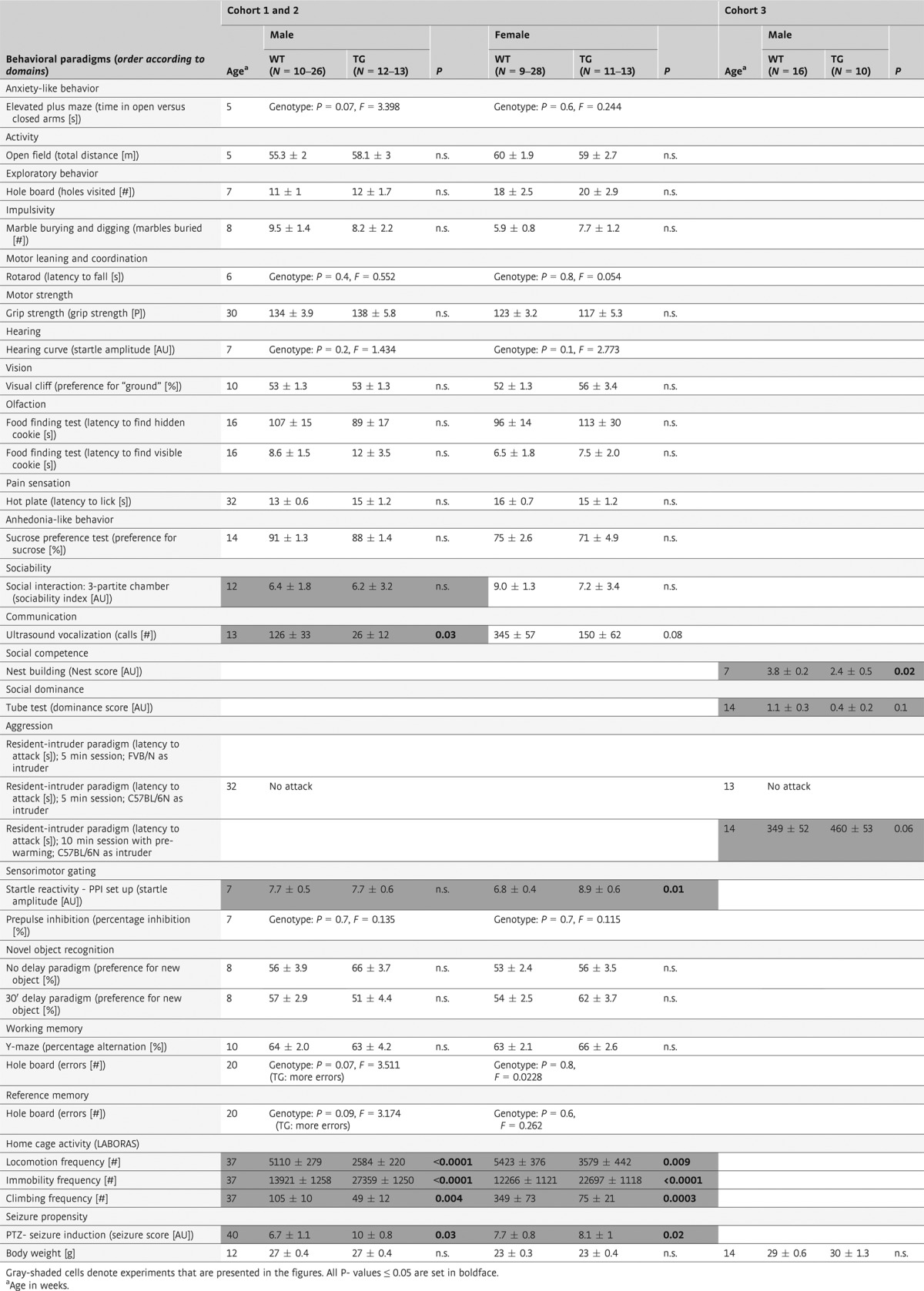

**Figure 1:**
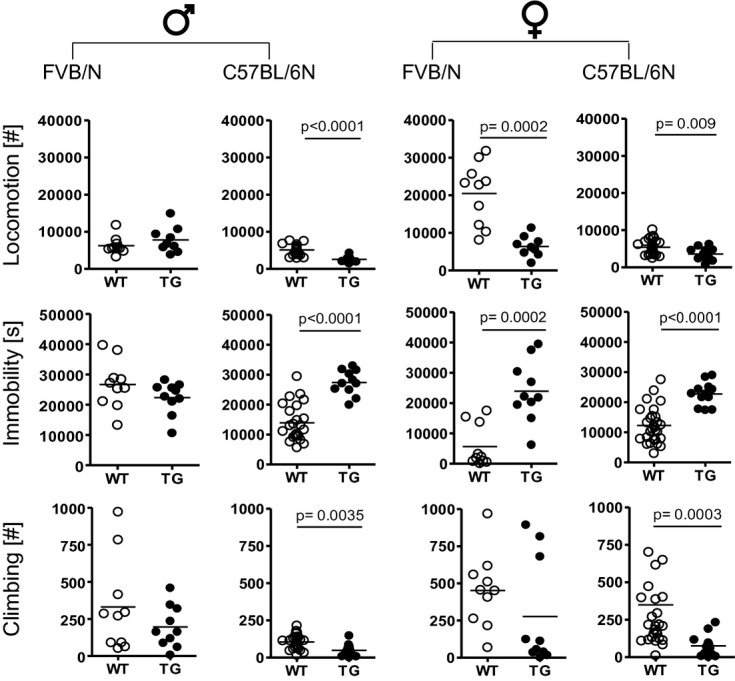
Spontaneous home cage activity of FVB/N and C57BL/6N mice of both genders is modulated by mild Mecp2 overexpression. Results for male and female mice of both strains are presented. With the exception of male FVB/N mice, locomotion, immobility and climbing reveal similar TG effects across genders and genetic backgrounds. *N* = 10–28; mean ± s.e.m. given.

We wondered whether subtle behavioral differences upon mild overexpression of *Mecp2* are perhaps not captured by the usual behavioral test battery. Therefore, spontaneous home cage behavior was monitored continuously overnight using LABORAS™. Indeed, we found significant differences between TG and WT mice. There was an overall tendency of reduced locomotion, including climbing, and increased immobility in TG as compared to WT, noticeable across strains and genders, except for male FVB/N mice that failed to show significant changes in locomotion (Fig 1).

### Male *Mecp2* transgenic mice of both genetic backgrounds exhibit altered territorial social behavior and aggression as compared to their WT littermates

**Figure 2:**
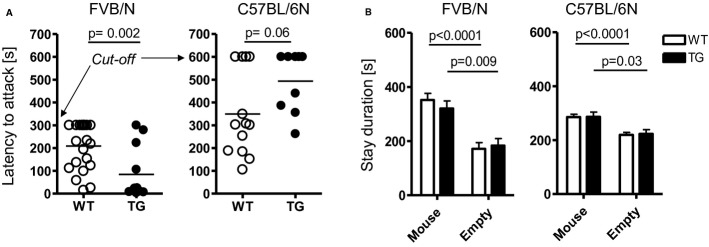
Territorial aggressive behavior in male mice is influenced by Mecp2 overexpression and genetic background. A Latency to attack in the resident-intruder test is significantly reduced in male FVB/N TG mice but increased in C57BL/6N. Note the different cut-off for the 2 strains. B Sociability testing in the tripartite chamber reveals a highly significant preference of male mice, independent of the genetic background, for a stranger mouse as compared to an object (empty cage). Data information: *N* = 10–24; mean ± s.e.m. given.

Based on the unexpected observation that mild overexpression of *Mecp2* leads to increased territorial aggressive behavior of male TG versus WT FVB/N mice when exposed to FVB/N intruders (Bodda *et al*, 2013), we started here a series of new experiments on the resident-intruder paradigm: (1) We aimed at testing reproducibility of this phenomenon in FVB/N mice exposed to younger intruder males of C57BL/6N background, that is, *per se* less aggressive intruders (Mineur & Crusio, 2002; Pugh *et al*, 2004). This experiment yielded similar results (Fig 2A), that is, increased territorial aggression in TG FVB/N. (2) We were interested to see whether another genetic background, C57BL/6N, would modify the effect of *Mecp2* overexpression. However, using the same resident–intruder protocol as in FVB/N males, no attack by C57BL/6N males was observed (Table 1b). As male C57BL/6N mice are generally less aggressive compared to male FVB/N (Mineur & Crusio, 2002; Pugh *et al*, 2004), the resident–intruder protocol had to be slightly modified by extending the cut-off time to 10 min and increasing the basal level of aggression using warming (Greenberg, 1972; Gaskill *et al*, 2012). Even though the necessary increase in surrounding temperature (<38°C) is far below the temperature used to test pain sensitivity (hot plate test; temperature set to 55°C), we note here that the pain threshold in TG and WT mice was identical (Table 1b). Prior to testing, the home cage of the resident mouse was placed under a heat-emitting red lamp for 20 min to obtain mild prewarming. Further, during testing, the home cage of the resident was positioned on a warming pad (set to 38°C). Surprisingly and opposite to FVB/N mice, the attack latency of TG C57BL/6N was longer, pointing to reduced territorial aggression as compared to WT littermates. In fact, within the 10 min observation, 30% of WT and 55% of TG residents of the C57BL/6N background did not attack the intruder at all (Fig 2A). In contrast to the clear alteration of territorial aggression in TG versus WT male mice of both strains, the sociability test (mouse preferred over empty cage) revealed entirely normal behavior, indistinguishable between TG and WT (Fig 2B).

**Figure 3:**
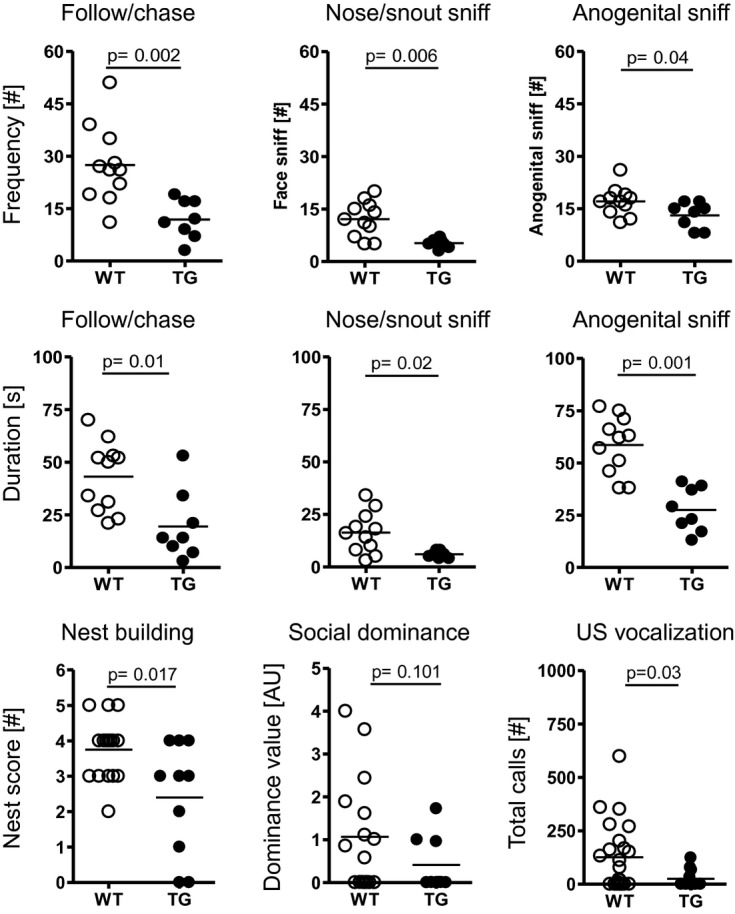
Male TG C57BL/6N mice show reduced territorial social interaction as well as inferior social competence. Upper 2 rows: Frequency and duration of determinants of territorial social interaction, that is, follow/chase behavior, nose/snout sniff and anogenital sniff, are consistently reduced in TG carriers. Lower row: TG mice are inferior in nest building, social dominance behavior and ultrasound vocalization. *N* = 10–23; mean ± s.e.m. given.

The unforeseen result of reduced aggression in male TG C57BL/6N prompted us to investigate the pattern of their territorial behavior during the resident–intruder test. We measured frequency and duration of agonistic encounters of the resident mouse for the first 3 min upon introducing the intruder. Whereas the latency to initiate the first contact was comparable between male WT and TG C57BL/6N mice (8.5 ± 1.3 s versus 10.7 ± 1.9 s; *P* = 0.35), TG mice exhibited lower frequency and duration of follow/chase behavior, as well as sniffing of facial and anogenital areas of the intruder mice than their WT littermates (Fig 3).

Since this territorial behavioral pattern of male C57BL/6N TG mice indicated somewhat reduced social interest, we hypothesized that these mice might also display other signs of changed social interest/competence. Indeed, nest building capacity/quality, social dominance measured by the tube test, and ultrasound vocalization in response to an anesthetized female intruder, revealed inferiority or at least a strong tendency thereof in TG animals (Fig 3). Together, these data indicate that also in *Mecp2* overexpressing mice of C57BL/6N background, territorial aggressive behavior is a central target phenotype. In these intrinsically non-aggressive mice, however, the direction of change is exactly opposite to the FVB/N strain and accompanied by reduced social interest and competence.

### Seizure propensity is increased upon mild *Mecp2* overexpression, independent of genetic background and gender

**Figure 4:**
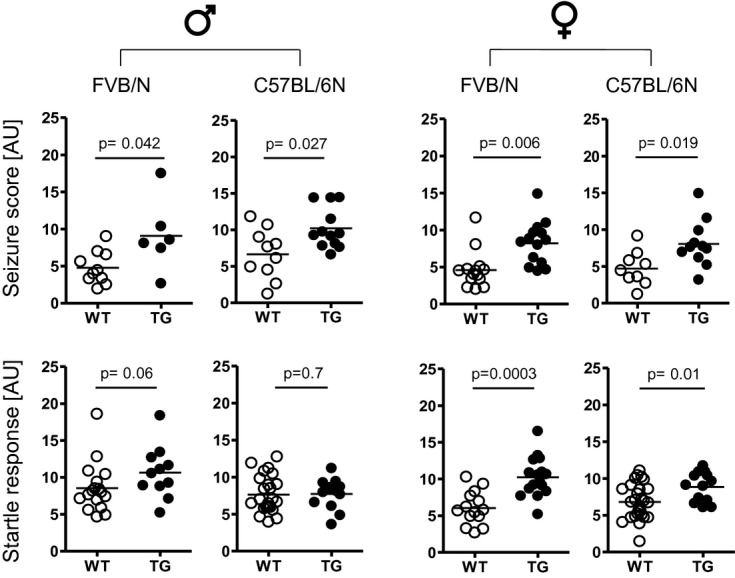
Pentylenetetrazole-induced seizure propensity is increased upon mild Mecp2 overexpression independent of strain and gender, whereas the startle response is augmented in females only. Upper row: Higher seizure scores are found in TG carriers across gender and genetic backgrounds. *N* = 6–14; Lower row: Significant increase in the startle response is observed only in female mice of both genetic backgrounds. *N* = 11–28; mean ± s.e.m. given.

A common characteristic neurological phenotype found in different mouse models of mutant *Mecp2,* ranging from complete *loss-of-function* to overexpression, is epileptic seizures (Guy *et al*, 2001; Shahbazian *et al*, 2002; Collins *et al*, 2004; Chahrour & Zoghbi, 2007). We previously reported in male FVB/N TG mice a higher susceptibility to seizure induction by the GABA_A_ receptor antagonist, pentylenetetrazole (PTZ) (Bodda *et al*, 2013). This work could now be expanded to female FVB/N TG as well as to male and female C57BL/6N TG mice. Indeed, PTZ-induced seizure propensity is increased across genetic backgrounds and genders (Fig 4), pointing to a strong overall phenotypical consequence of mild *Mecp2* overexpression.

### The startle response to acoustic stimulation is augmented upon mild *Mecp2* overexpression in female mice of both genetic backgrounds

Deletion of *Mecp2* in GABAergic neurons resulted in reduction in the startle response to acoustic stimuli of 120db (Chao *et al*, 2010), but nothing has been known regarding a potential influence of mild *Mecp2* overexpression on startling. Since *Mecp2* TG mice of both genetic backgrounds exerted an exaggerated seizure reaction to a GABA_A_ receptor antagonist, PTZ, we wondered whether mild overexpression of *Mecp2* might also alter the startle reflex and/or the prepulse inhibition of the startle response (PPI). Indeed, we found enhanced startling in female but not male *Mecp2* TG mice of both FVB/N and C57BL/6N background (Fig 4). No changes in the percentage of PPI were observed in any strain or gender (Table 1a,b). Similar findings that the startle response at 120db does not necessarily affect overall PPI were reported in the context of a study on mouse inbred strain differences (Paylor & Crawley, 1997).

### *MECP2* SNP distribution is comparable in healthy and schizophrenic individuals

**Figure 5:**
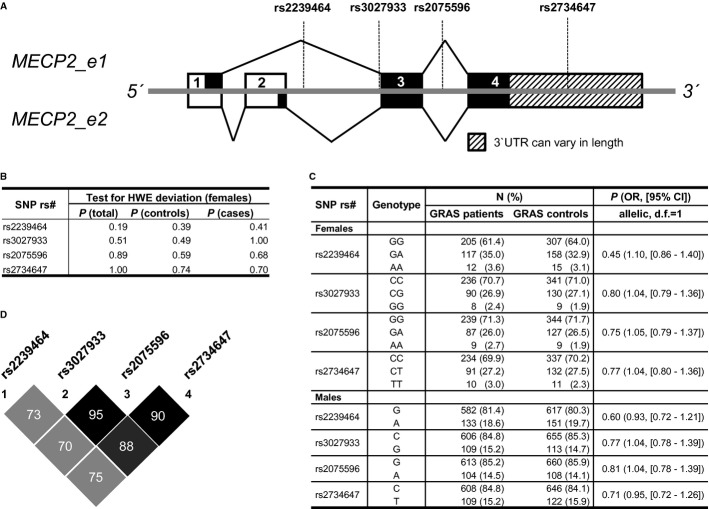
Basic genetics of *MECP2*: Gene structure, Hardy-Weinberg statistics, linkage disequilibrium, and case-control analysis in schizophrenic (GRAS) patients and healthy individuals. A Schematic overview of MECP2 isoforms e1 and e2, including SNP positions. Digits depict exon numbers, solid black lines exon usage for the respective isoform. Dashed lines denote SNP positions. Black fillings in boxes denote coding sequence, isoform-specific in exons 1 and 2. B Test for deviation from Hardy-Weinberg-Equilibrium (HWE) in females only due to X-chromosomal location of *MECP2*. C Case–control association analysis - separate for both genders - reveals similar distribution of SNPs in patients and controls. D Linkage disequilibrium for all included GRAS patients and controls.

Since our mouse studies revealed aggressive social behavior as a central phenotype modulated by subtle *Mecp2* overexpression, we started a hypothesis-driven analysis on subjects of the GRAS (Göttingen Research Association for Schizophrenia) data collection (Ribbe *et al*, 2010). Genotyping for association analyses was performed for 4 SNPs located in the X-chromosomal *MECP2* gene with reasonably high minor allele frequency (Fig 5A). At first, a potential genetic risk condition of the selected SNPs for schizophrenia was assessed: A case–control study comparing allele frequencies of the 4 genotyped SNPs rs2239464, rs3027933, rs2075596, and rs2734647 in 1052 GRAS patients versus 1248 healthy controls was performed separately for men and women. All markers fulfilled Hardy–Weinberg criteria, and no significant associations with diagnosis were detected (Fig 5B,C). Due to strong linkage disequilibrium (LD) between markers, only rs2239464 and rs2734647 were considered for the phenotype-based genetic association study (PGAS, inclusion criterion *r*² < 0.8) (Fig 5D).

### Normal genetic variation of *MECP2* influences aggression and impulsivity in man

We next selected aggression-related variables from the GRAS phenotypical data collection and tested their association with the 2 selected *MECP2* SNPs separately in men and women. *Poor impulse control* and a further trait reflecting behavioral expression of impulsive aggression (*excitement*) were chosen. For both, SNP rs2734647 in the 3′UTR (Table 2) and for SNP rs2239464 (Table 3), significant associations with these traits were found in men, with C carriers and G carriers (the major alleles), respectively, being more aggressive. Sociodemographic and clinical measures potentially confounding these results did not differ between genotype groups. The nominally significant result for years of education in males (SNP rs2734647) was accounted for by including it as a covariate in all models with target measures as dependent variables. In contrast, for women, no statistically significant results were obtained. The phenotype–genotype relationships could be reproduced in a small independent sample of schizophrenic men (Table 4). Taken together, an association of *MECP2* genotypes with readouts of aggression was found in two independent cohorts of men.

**Table 2:** Phenotype comparison of GRAS patients by *MECP2* SNP rs2734647 genotypes

Males (GRAS sample)^a^	C	T	*P*-value (*F/Z/*χ^*2*^ value)^b^
Target variables^C^	*N* = 491 to 608	*N* = 73 to 109	
Poor impulse control, mean ± s.d. [range]	1.72 ± 1.12 [1 to 6]	1.39 ± 0.76 [1 to 4]	**0.0001** (*F* = 14.65)
Excitement, mean ± s.d. [range]	2.05 ± 1.30 [1 to 7]	1.82 ± 1.13 [1 to 6]	**0.034** (*F* = 4.52)
Control variables			
Sociodemographic variables			
Age (at examination), years, mean ± s.d. [range]	37.40 ± 12.09 [17 to 78]	37.02 ± 11.99 [21 to 71]	0.736 (*Z = −*0.34)
Education, years, mean ± s.d. [range]^d^	12.11 ± 3.10 [0 to 24]	11.32 ± 2.48 [8 to 18]	**0.020** (*Z = −*2.33)
Unemployment, No. (%)	107 (17.8)	22 (21.0)	0.446 (χ^2^ = 0.58)
Clinical variables			
PANSS general score, mean ± s.d. [range]^e^	30.57 ± 10.82 [15 to 81]	31.88 ± 9.73 [16 to 55]	0.542 (*Z *=* *−0.61)
PANSS negative score, mean ± s.d. [range]	18.04 ± 7.67 [7 to 46]	19.23 ± 7.31 [7 to 40]	0.070 (*Z = *−1.81)
PANSS positive score, mean ± s.d. [range]^f^	11.61 ± 5.25 [6 to 34]	11.39 ± 5.15 [6 to 30]	0.667 (*Z = *−0.43)
Cognition composite score, mean ± s.d. [range]^g^	0.09 ± 0.84 [−2.4 to 2.2]	0.00 ± 0.84 [−2.1 to 1.7]	0.543 (*F* = 0.37)
Chlorpromazine equivalents, mean ± s.d. [range]	688 ± 644 [0 to 4511]	725 ± 734 [0 to 6324]	0.394 (*Z = −*0.85)
GAF score, mean ± s.d. [range]	45.48 ± 16.35 [5 to 90]	46.94 ± 15.45 [15 to 85]	0.375 (*Z* = −0.89)

Females (GRAS sample)^a^	CC	CT	TT	*P*-value (*F/Z/*χ^*2*^ value)^b^
Target variables^c^	*N* = 194 to 234	*N* = 73 to 91	*N* = 8 to 10	
Poor impulse control, mean ± s.d. [range]	1.73 ± 1.15 [1 to 7]	1.57 ± 0.94 [1 to 5]	1.60 ± 1.07 [1 to 4]	0.729 (*F* = 0.31)
Excitement, mean ± s.d. [range]	2.11 ± 1.30 [1 to 7]	1.93 ± 1.31 [1 to 7]	1.50 ± 0.53 [1 to 2]	0.277 (*F* = 1.29)
Control variables				
Sociodemographic variables				
Age (at examination), years, mean ± s.d. [range]	43.41 ± 12.90 [18 to 76]	41.82 ± 12.47 [19 to 72]	43.52 ± 12.10 [20 to 58]	0.657 (χ^2^* *= 0.84)
Education, years, mean ± s.d. [range]^d^	12.61 ± 3.36 [8 to 27]	12.14 ± 2.83 [8 to 21]	12.15 ± 3.76 [8 to 19]	0.750 (χ^2^* *= 0.58)
Unemployment, No. (%)	25 (10.9)	12 (13.6)	4 (40.0)	0.176 (χ^2^* *= 1.83)
Clinical variables				
PANSS general score, mean ± s.d. [range]^e^	33.04 ± 12.10 [15 to 69]	32.38 ± 12.25 [15 to 72]	31.30 ± 10.64 [19 to 54]	0.872 (χ^2^* *= 0.28)
PANSS negative score, mean ± s.d. [range]	18.13 ± 8.50 [7 to 44]	17.82 ± 8.11 [7 to 36]	19.30 ± 9.36 [7 to 39]	0.904 (χ^2^* *= 0.20)
PANSS positive score, mean ± s.d. [range]^f^	12.25 ± 6.03 [6 to 32]	11.41 ± 5.25 [6 to 30]	11.40 ± 4.40 [6 to 18]	0.650 (χ^2^* *= 0.86)
Cognition composite score, mean ± s.d. [range]^g^	−0.02 ± 0.83 [−2.0 to 1.9]	−0.19 ± 0.90 [−2.0 to 2.0]	0.08 ± 0.83 [−1.5 to 1.1]	0.090 (*F* = 2.43)
Chlorpromazine equivalents, Mean ± s.d. [range]	677 ± 839 [0 to 7375]	608 ± 685 [0 to 4375]	640 ± 512 [150 to 1680]	0.614 (χ^2^* *= 0.98)
GAF score, mean ± s.d. [range]	45.44 ± 19.07 [12 to 90]	47.07 ± 18.60 [8 to 90]	48.80 ± 14.73 [25 to 70]	0.472 (χ^2^ = 1.50)

All *P *≤ 0.05 are set in boldface.

GAF, global assessment of functioning

a Due to missing data, sample sizes vary.

b For statistical methods, Mann–Whitney *U/*Kruskal–Wallis test (women) or Chi-square tests and for models including covariates ANCOVAs (target variables and cognition composite score) were used.

c ANCOVA with education, age at examination, negative symptoms (PANSS) and medication status (chlorpromazine equivalent) as covariates.

d Years spent in education system; patients currently in school or educational training included (score 0).

e Item 14 (target variable) excluded from sum score.

f Item 4 (target variable) excluded from sum score.

g ANCOVA with age, negative symptoms (PANSS) and medication status (chlorpromazine equivalent) as covariates.

**Table 3:** Phenotype comparison of GRAS patients by *MECP2* SNP rs2239464 genotypes

Males (GRAS sample)^a^	G	A	*P*-value (*F/Z/*χ^*2*^ value)^b^
Target variables^c^	*N* = 469 to 582	*N* = 94 to 134	
Poor impulse control, mean ± s.d. [range]	1.72 ± 1.12 [1 to 6]	1.44 ± 0.81 [1 to 4]	**0.001** (*F* = 10.84)
Excitement, mean ± s.d. [range]	2.05 ± 1.31 [1 to 7]	1.83 ± 1.11 [1 to 6]	0.056 (*F* = 3.67)
Control variables			
Sociodemographic variables			
Age (at examination), years, mean ± s.d. [range]	37.31 ± 12.07 [17 to 78]	37.27 ± 12.12 [21 to 71]	0.918 (*Z* = −0.10)
Education, years, mean ± s.d. [range]^d^	12.10 ± 3.10 [0 to 24]	11.50 ± 2.52 [8 to 20]	0.063 (*Z* = −1.86)
Unemployment, No. (%)	101 (17.6)	28 (21.5)	0.294 (*χ*^2^ = 1.10)
Clinical variables			
PANSS general score, mean ± s.d. [range]^e^	31.71 ± 10.84 [15 to 81]	31.36 ± 9.83 [16 to 55]	0.966 (Z = −0.04)
PANSS negative score, mean ± s.d. [range]	18.13 ± 7.66 [7 to 46]	18.61 ± 7.48 [7 to 40]	0.420 (*Z =* −0.81)
PANSS positive score, mean ± s.d. [range]^f^	11.67 ± 5.29 [6 to 34]	11.20 ± 4.99 [6 to 30]	0.358 (*Z =* −0.92)
Cognition composite score, mean ± s.d. [range]^g^	0.09 ± 0.83 [−2.4 to 2.2]	0.05 ± 0.88 [−2.1 to 1.9]	0.842 (*F* = 0.40)
Chlorpromazine equivalents, mean ± s.d. [range]	691 ± 635 [0 to 4511]	688 ± 700 [0 to 6324]	0.936 (*Z =* −0.08)
GAF score, mean ± s.d. [range]	45.11 ± 16.26 [5 to 90]	45.27 ± 15.7 [15 to 85]	**0.045** (*Z =* −2.00)

Females (GRAS sample)^a^	GG	GA	AA	*P*-value (*F/Z/*χ^*2*^ value)^b^
Target variables^c^	*N* = 173 to 206	*N* = 94 to 117	*N* = 10 to 13	
Poor impulse control, mean ± s.d. [range]	1.74 ± 1.18 [1 to 7]	1.62 ± 0.99 [1 to 5]	1.23 ± 0.60 [1 to 3]	0.243 (*F* = 1.42)
Excitement, mean ± s.d. [range]	2.12 ± 1.29 [1 to 7]	1.98 ± 1.31 [1 to 7]	1.62 ± 1.12 [1 to 5]	0.416 (*F* = 0.88)
Control variables				
Sociodemographic variables				
Age (at examination), years, mean ± s.d. [range]	43.65 ± 13.11 [18 to 76]	42.01 ± 12.15 [19 to 72]	42.66 ± 12.37 [20 to 58]	0.665 (χ^2^* *= 0.82)
Education, years, mean ± s.d. [range]^d^	12.66 ± 3.32 [8 to 27]	12.22 ± 3.05 [8 to 21]	11.96 ± 3.28 [8 to 19]	0.538 (χ^2^* *= 1.24)
Unemployment, No. (%)	20 (10.0)	17 (14.5)	4 (30.8)	0.056 (χ^2^* *= 5.77)
Clinical variables				
PANSS general score, mean ± s.d. [range]^e^	32.92 ± 12.42[15 to 69]	33.11 ± 11.79 [15 to 72]	30.23 ± 11.42 [19 to 54]	0.690 (χ^2^* *= 0.74)
PANSS negative score, mean ± s.d. [range]	18.07 ± 8.49 [7 to 44]	18.20 ± 8.37 [7 to 39]	17.62 ± 8.48 [7 to 39]	0.961 (χ^2^* *= 0.08)
PANSS positive score, mean ± s.d. [range]^f^	12.28 ± 5.99 [6 to 32]	11.74 ± 5.55 [6 to 32]	10.00 ± 4.26 [6 to 18]	0.440 (χ^2^* *= 1.64)
Cognition composite score, mean ± s.d. [range]^g^	0.01 ± 0.84 [−2.0 to 1.9]	−0.20 ± 0.87 [−2.0 to 2.0]	0.03 ± 0.75 [−1.5 to 1.4]	**0.049** (*F* = 3.06)
Chlorpromazine equivalents, mean ± s.d. [range]	671 ± 852[0 to 7375]	638 ± 702 [0 to 4370]	650 ± 504 [150 to 1680]	0.811 (χ^2^* *= 0.41)
GAF score, mean ± s.d. [range]	46.07 ± 19.12 [15 to 90]	45.13 ± 18.87 [8 to 90]	51.38 ± 12.35 [25 to 70]	0.323 (χ^2^* *= 2.26)

All *P* ≤ 0.05 are set in boldface.

a Due to missing data, sample sizes vary.

b For statistical methods, Mann–Whitney *U/*Kruskal–Wallis test (women) or Chi-square tests and for models including covariates ANCOVAs (target variables and cognition composite score) were used.

c ANCOVA with education, age at examination, negative symptoms (PANSS) and medication status (chlorpromazine equivalent) as covariates.

d Years spent in education system; patients currently in school or educational training included (score 0).

e Item 14 (target variable) excluded from sum score.

f Item 4 (target variable) excluded from sum score.

g ANCOVA with age, negative symptoms (PANSS) and medication status (chlorpromazine equivalent) as covariates.

**Table 4:** Phenotype comparison of replication sample patients by *MECP2* SNPs rs2734647 and rs2239464

	rs2734647 genotypes		*P*-value (*Z/*χ^*2*^ value)^a^
Males (replication sample)	C	T	
Target variables^b^	*N* = 322	*N* = 63	
Poor impulse control, mean ± s.d. [range]	3.16 ± 1.91 [1 to 7]	2.86 ± 1.93 [1 to 6]	**0.05** (*Z =* −1.64)
Excitement, mean ± s.d. [range]	3.79 ± 1.60 [1 to 7]	3.52 ± 1.56 [1 to 7]	0.09 (*Z =* −1.33)
Control variables			
Age (at examination), years, mean ± s.d. [range]	35.34 ± 10.91 [19 to 65]	36.63 ± 10.80 [18 to 67]	0.16 (*Z *=* *−1.01)
Education rating,% low, intermediate, high education level	47.8%, 22.4%, 29.8%	42.9%, 30.2%, 27.0%	0.21 (χ^*2*^* *= 1.78)

	rs2239464 genotypes		*P*-value (*Z/*χ^*2*^ value)^a^
Males (replication sample)	G	A	
Target variables^b^	*N* = 308	*N* = 77	
Poor impulse control, mean ± s.d. [range]	3.19 ± 1.92 [1 to 7]	2.82 ± 1.89 [1 to 6]	**0.03** (*Z =* −1.91)
Excitement, mean ± s.d. [range]	3.83 ± 1.60 [1 to 7]	3.42 ± 1.58 [1 to 7]	**0.02** (*Z =* −2.07)
Control variables			
Age (at examination), years, mean ± s.d. [range]	35.28 ± 11.01 [19 to 65]	36.66 ± 10.40 [18 to 67]	0.12 (*Z = *−1.23)
Education rating,% low, intermediate, high education level	48.7%, 22.1%, 29.2%	40.3%, 29.9%, 29.9%	0.14 (χ^2^ = 2.53)

All *P* ≤ 0.05 are set in boldface.

a For statistical methods, Mann–Whitney *U* or Chi*-*square tests were used (all *P*-values one-sided).

b Mann–Whitney *U*-tests with standardized residuals from linear regression with target variables as dependent variables and age at examination and negative symptoms as independent variables.

### SNP rs2734647 in the 3′UTR affects miR-511 binding and gene expression

**Figure 6:**
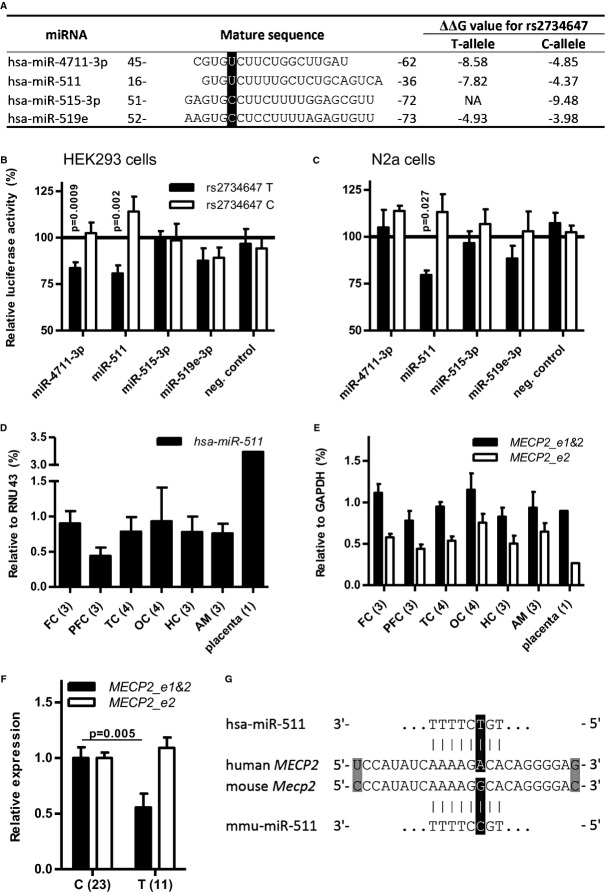
SNP rs2734647 in the 3'UTR of MECP2: Search for mechanistic insight. A Human miRNAs predicted to bind to the *MECP2* 3′UTR in an rs2734647 allele-specific manner. The bases corresponding to the SNP position are black-shadowed. Numbers left and right of the nucleotide sequence refer to its base-pair position within the miRNA sequence. B, C Luciferase assay results showing relative luciferase activity in HEK293 and N2a cells after co-transfection of candidate miRNAs with phRL-TK rs2734647C, or rs2734647T, respectively; mean ± s.e.m.;*N* = 7 (refers to biological replicates) for all conditions. Statistical significance was calculated relative to the non-transfection control (100%). D, E Relative expression of hsa-miR-511 and of MECP2 isoform 2 (*MECP2 e2*) or both isoforms (*MECP2 e1*&*2*) in aggression/impulsivity – relevant brain areas: FC=frontal cortex, PFC, prefrontal cortex; TC, temporal cortex; OC, occipital cortex; HC, hippocampus; AM, amygdala; as well as in placenta (*N* = 1) as control tissue. Numbers of individual brains included in the analysis are given in brackets; mean ± s.e.m. F Relative expression of *MECP2 e2* or *MECP2 e1&2* in peripheral blood mononuclear cells (PBMC) of male patients dependent on rs2734647 genotype; N numbers of individuals in brackets; mean ± s.e.m. G Alignment of human and mouse *MECP2* 3′UTRs around rs2734647 SNP position (black-shadowed) and human and mouse miR-511, illustrating a perfect species-specific seed match; hsa-miR-511 perfectly matches the human *MECP2* 3′UTR in case of rs2734647 T. Additional mismatches are gray-shadowed.

To examine possible functional implications of SNP rs2734647 in the 3′UTR on microRNA (miR)-dependent regulation of gene expression, *in silico* analyses were performed using TargetScan (Release 6.2) (Lewis *et al*, 2005; Grimson *et al*, 2007) and PITA (Kertesz *et al*, 2007), resulting in the prediction of 4 miRs with seed binding sites comprising the SNP rs2734647 position (i.e., c.*3638A>G). Allele-specific differences of the predicted ΔΔG values, indicating strength of miR binding, are summarized in Fig 6A. While miR-4711-3p and miR-511 are predicted to show preferential binding in case of the presence of the T-allele, miR-515-3p has a strong negative ΔΔG only in case of the C-allele, and miR-519e lacks a strong allele preference. *In vitro* luciferase assays using HEK293 cells revealed significantly reduced luciferase activity in case of co-transfection of the plasmid carrying the T-allele with both miR-4711-3p and miR-511 (Fig 6B). Co-transfection with miR-515-3p or miR-519e did not lead to luciferase activity reduction for either rs2734647 T or C (Fig 6B). In luciferase assays using N2a cells, the positive result was only replicated for miR-511 (Fig 6C). In summary, these data strongly suggest miR-511 as rs2734647 genotype-dependent candidate for *MECP2* regulation in humans. For the sake of completeness, we determined endogenous expression of miR-511 in the cell lines used for transfection. Whereas mmu-miR-511 levels were under the detection limit in N2a cells, hsa-miR-511 was clearly expressed in HEK293 cells (3.59 × 10^−3^). However, since we always used negative, that is, non-miR-transfected controls, Luciferase assay results are unlikely to have been affected.

### miR-511 is expressed in aggression-relevant humanbrain regions

Since miR-511 seems to be an important modulator of rs2734647 genotype-dependent *MECP2* expression in humans, we asked whether this miR would be detectable in brain regions relevant for impulsivity and aggression (frontal and prefrontal cortex, temporal cortex, occipital cortex, hippocampus, and amygdala). Placenta was used as developmental control tissue. In all regions examined, miR-511 expression was found (Fig 6D). Importantly, the same holds true for *MECP2* using primers amplifying either *MECP2*_e2 only or both isoforms (Fig 6E). In contrast, miR-4711-3p expression was not detected (data not shown), at least questioning a role of this miR for MECP2 regulation in the adult human brain. In the adult C57BL/6N versus FVB/N mouse brain, the expression of miR-511 was low and comparable between strains in cortex (2.39 × 10^−5^ versus 3.71 × 10^−5^), hippocampus (6.45 × 10^−5^ versus 3.78 × 10^−5^), and cerebellum (2.22 × 10^−5^ versus 2.36 × 10^−5^). Strong expression was found in embryonic tissue (C57BL/6N embryo head E17: 1.83 × 10^−3^; embryo body: 2.26 × 10^−3^), and highest in cultured microglia (13.02 × 10^−3^).

### Peripheral blood mononuclear cells (PBMC) of male rs2734647 T-carriers show lower *MECP2* expression

Since miR-511 was shown to be expressed in dendritic cells and macrophages (Tserel *et al*, 2011), we investigated the hypothesized allele-specific downregulation of *MECP2* expression in PBMC of male subjects. Indeed, a significantly lower expression in T carriers versus C carriers (amounting to around 50%) was detected with primers amplifying both *MECP2* isoforms, whereas *MECP2_e2* alone showed no expression difference dependent on the rs2734647 genotype (Fig 6F). This result may indicate that in T carriers mainly the *MECP2_e1* isoform is affected, at least in PBMC, potentially related to isoform-specific different lengths of the *MECP2* 3′UTR (Coy *et al*, 1999).

### Sequence variation of miR-511 in mouse and man underlines the importance of an interaction with the *MECP2* 3′UTR

A perfect seed match of human hsa-miR-511 with the *MECP2* 3′UTR in carriers of rs2734647-T is the most likely reason for the difference in luciferase activity in contrast to the C-allele, which results in a mismatch in the seed binding region. C is the conserved ancestral allele found in multiple other mammalian species, including mouse. Strikingly, the seed sequence of mouse mmu-miR-511 differs from hsa-miR-511 exactly regarding the nucleotide complementary to the SNP position (Fig 6G). Thus, mmu-miR-511 shows an ideal seed match to the mouse 3′UTR, whereas hsa-miR-511 perfectly fits to the human 3′UTR carrying the T-allele. This observation may emphasize the importance of the miR-511 interaction with *MECP2*. Notably, screening of available data from Jackson Laboratories (http://www.jaxlab.org) revealed that the two mouse lines employed here are not polymorphic for the *Mecp2* 3′UTR allele or the miR-511 sequence. It would, however, be interesting to investigate whether non-inbred mice are polymorphic for the respective alleles.

## Discussion

The present study shows that mildly increased *Mecp2/MECP2* expression leads to alterations in male social aggression. Using C57BL/6N versus FVB/N mice (with their known inherent strain differences in aggressive behavior) (Mineur & Crusio, 2002; Pugh *et al*, 2004) as models, we demonstrate that the direction of change upon mild *Mecp2* overexpression in this behavioral target domain is subject to modification by the genetic background. In other words, the resulting lower or higher social aggression depends on the basic genetic make-up of a particular subject. Hints of *MECP2* influencing aggressive behavior could also be shown in two independent cohorts of schizophrenic men, with a polymorphism in the 3′UTR of the gene co-determining both the level of *MECP2* expression as well as of aggression. The genotype-dependent expression difference in men found in PBMC (around 50%) is in the range of the transgenic overexpression in both mouse strains, emphasizing the physiological significance of these findings as well as of our mouse models for studying behavioral consequences of a normal “*Mecp2* dose range”.

Even though in both schizophrenia samples, higher expression of *MECP2* (3′UTR SNP rs2734647 C carriers lack suppressibility by miR-511) was associated with higher aggression, it has to be considered that also humans are not an isogenic population. One might thus, in analogy to our observation in different mouse strains, predict a human population with reduced aggression as suggested by the C57BL/6N data (i.e., a bimodal aggression distribution when combining different genetic backgrounds).

Although the results for women show a similar tendency for poor impulse control and excitement as aggression readouts, they are far from reaching significance, likely due to the smaller number of individuals, but certainly also to the fact that *MECP2* is X chromosomal. Homozygous T carriers in the female sample are therefore expectedly very rare.

In our translational PGAS approach, we had the chance to explore the aggression association of *MECP2* genotypes in a phenotypically very well-characterized large sample of schizophrenic individuals (Ribbe *et al*, 2010), and we replicated the association findings in a second, independent cohort of schizophrenic men. Therefore, we cannot state with certainty at this point that the phenotype association holds true in the same way for healthy individuals. Nevertheless, apart from the supporting data obtained for healthy mice, it has to be emphasized that aggression is not a specific or unique symptom in schizophrenia. Also, the range of aggressive features in the whole GRAS patient sample follows a normal distribution, extending from very low to very high aggression scores.

Along the same lines, the case–control study presented here, including 1052 cases and 1248 controls, fails to attribute to *MECP2* any schizophrenia risk gene role. In some contrast, a recent study reported in a Han Chinese population an association of rs2734647 C with the disease (498 cases versus 2025 controls, replicated in 1027 cases versus 1005 controls) (Wong *et al*, 2013). Although we did not even find a respective trend (Fig 5C), we cannot entirely exclude limited power of our case–control approach. On the other hand, the association might well be population-specific. In any case, a potential risk gene status, even if confirmed by future GWAS including X-chromosomal genotypes, will not be dramatic considering the large number of individuals needed for its demonstration. Instead, *MECP2* is most likely disease-independently involved in the regulation of a basic mammalian behavioral phenotype, that is, aggression. Interestingly, carriers of the minor allele of *MECP2* SNP rs2239464 were previously shown to have decreased cortical surface area in brain regions such as the cuneus (Joyner *et al*, 2009), which is associated with inhibitory control in patients with bipolar disorder (Haldane *et al*, 2008).

In further support of an association between *MECP2* and aggression, impulse control alterations in individuals with *MECP2/Mecp2* gene duplication syndrome have been reported (Ramocki *et al*, 2009), even though the findings of the present study are more relevant for the understanding of physiological gene-dose effects on social behavior. Importantly, Mecp2/MECP2 functions as transcriptional regulator targeting hundreds of other genes (Chen *et al*, 2003; Sun & Wu, 2006; Bird, 2008; Chahrour *et al*, 2008; Ben-Shachar *et al*, 2009; Wu *et al*, 2010). Thus, it is most likely a whole pattern of genes—directly or indirectly influenced by this regulator—that primes nuances of aggressive social behavior. As an example, changes in the expression of a Mecp2 regulated gene, *Prom1,* are associated with domestication and aggressive behavior in animals (Albert *et al*, 2012; Gopisetty *et al*, 2012). Furthermore, Mecp2 is known to control expression of brain-derived neurotrophic factor (Bdnf) (Martinowich *et al*, 2003), which in turn is involved in the regulation of aggression (Ito *et al*, 2011).

Aggression seems to be a strong target phenotype of mild *Mecp2* overexpression independent of the genetic background, since these expression changes did not lead to alterations in basic behavior, including motor, sensory and cognitive functions. Male and female TG mice of both the FVB/N and C57BL/6N genetic backgrounds displayed basic behavior comparable to their WT littermates. Apart from aggression, only home cage activity, seizure propensity and startle response were influenced by mild *Mecp2* overexpression in a fashion widely independent of the genetic background.

There have been reports on sexual dimorphism with respect to *Mecp2* expression and function in the brain. For instance, in amygdalae and ventromedial hypothalamus, male rats express less *Mecp2* as compared to females (Kurian *et al*, 2007, 2008). Furthermore, conditional knockout of *Mecp2* during amygdala development caused subtle modifications of juvenile play behavior in male but not female rats (Kurian *et al*, 2008). These findings may indicate a role of *Mecp2* in gender-specific modulation of behavior. In the present work, however, sexual dimorphism was consistently observed only with the startle response in a genotype and genetic background independent manner.

Even though the explicit situations are still unknown in which miR-511 regulated *MECP2/Mecp2* expression might be of particular physiological relevance, any kind of inflammation in the brain for instance could play a pivotal role, considering the relatively high expression found here in mouse microglia. The distinct suppression of *MECP2* expression by miR-511 in SNP rs2734647-T carriers reported here may even be considered as a future treatment target in *MECP2* gene duplication syndrome. In any case, the high conservation of the interaction between miR-511 and *MECP2* in both mouse and man makes a specific significance of their interplay very likely. This significance is further supported by the here demonstrated co-expression of *MECP2* and miR-511 in human brain areas pivotal for aggression and impulsivity regulation (Brower & Price, 2001; Horn *et al*, 2003; Berlin *et al*, 2004; Bauman *et al*, 2006; Zetzsche *et al*, 2007; Siever, 2008; Whelan *et al*, 2012). Interestingly, miR-511 expression was found here also in different mouse brain areas, with levels comparable across both genetic backgrounds.

To conclude, MECP2/Mecp2 has been shown here to be a regulator of social aggressive behavior in mouse and man, with the genetic background playing an important modifier role.

## Materials and Methods

### Mice

All mouse experiments have been approved by the Animal Care and Use Committee of Lower Saxony, Oldenburg, Germany. The generation of *Mecp2*^*WT_EGFP*^ TG mice with a 1.4–1.5-fold *Mecp2* overexpression on FVB/N background has been described in detail previously (Bodda *et al*, 2013). Briefly, a bacterial artificial chromosome (BAC) clone, pBAC_B22804, containing 120 Kb of murine genomic fragment with the intact *Mecp2* gene and the flanking *Opsin1* and *Irak1* genes was used for generating the transgenic construct (Kifayathullah *et al*, 2010). To generate the pBAC_Mecp2WT_EGFP construct, the enhanced green fluorescent protein/kanamycin-resistant gene (*EGFP/kan*) cassette was PCR amplified using pEGFP1 vector as template with primers containing 50-bp flanking sequence from either side of the *Mecp2* stop codon. The endogenous stop codon was replaced by two glycine residues inframe between the Mecp2 protein and the EGFP protein to facilitate the two proteins to fold and function independently. The amplified *EGFP/Kan* cassette was electroporated into E.coli harboring the BAC clone and pGET recombination system, to facilitate the homologous recombination of *EGFP/Kan* cassette at the site of stop codon of *Mecp2*. The correct insertion of *EGFP/Kan* cassette after the recombination event into the BAC clone was confirmed by sequencing. The *Mecp2* flanking genes, *Opsin1* and *Irak1* were deleted from the modified BAC clone using additional BAC recombineering with zeocin selection cassette (containing the BAC homology arms and zeocin antibiotic marker gene driven by EM7 promoter from pSELECT vector) (InvivoGen, Toulouse, France), to avoid any additional phenotype arising from the overexpression of these genes. During the process of *Opsin1* deletion, a MluI restriction site was introduced into BAC clone. The final BAC construct pBAC_Mecp2WT_EGFP was linearized with MluI restriction enzyme and micro-injected into the male pronuclei of the fertilized mouse oocytes derived from the FVB/N strain. Next, the injected oocytes were transplanted into the uteri of the foster mothers. Genomic DNA isolated from tail biopsies was analyzed for the presence of the transgene by PCR. Because the transgene was not confirmed to be localized to the X chromosome, translational relevance with respect to modeling mosaicism resulting from X chromosome loss is limited.

To create a comparator congenic strain for behavioral analyses, *Mecp2* TG FVB/N mice were backcrossed for 10 generations to the C57BL/6N background. For experiments reported here, male and female *Mecp2*^*WT_EGFP*^ TG (hemizygous) and their WT littermates on either FVB/N or C57BL/6N backgrounds were used. Tail biopsies were taken before weaning to obtain genomic DNA for genotyping (Kifayathullah *et al*, 2010; Bodda *et al*, 2013). Western blot and qPCR analyses for TG expression estimation were performed as described previously (Bodda *et al*, 2013).

### Behavioral analyses

After weaning and during the whole period of behavioral testing, mice were housed individually in standard plastic cages (26.5 × 20 × 14 cm) and kept under temperature-controlled environment (21 ± 2°C) on 12 h light/dark cycle with food and water *ad libitum*, unless stated otherwise. Single housing was necessary since male FVB/N mice exhibited extremely aggressive behavior in a group-housed setting. In order to avoid housing differences as confounding variables, we decided to single-house all mice, independent of gender and strain. All experiments were conducted by investigators unaware of the genotype (“blinded”), during the light phase of the day (between 8:00 am and 6 pm, except for automated home cage behavioral assessment. Several independent cohorts of mice (genders and strains tested separately, starting at 5 weeks of age) were run through a battery of tests covering altogether basic behavioral, sensory, motor, cognitive and social functions (for overview see Table 1a,b). The order of tests was always oriented toward increasing invasiveness and performed as published in detail earlier (Jamain *et al*, 2008; El-Kordi *et al*, 2012; Bodda *et al*, 2013): Elevated plus maze (anxiety), open field (spontaneous activity), hole board (exploratory behavior), grip strength and rotarod (motor force, balance and coordination), marble burying (stereotypies and obsessive–compulsive behaviors), prepulse inhibition of the startle response (sensorimotor gating), hearing test (startle curve upon random presentation of stimulus intensities from 65 dB to 120 dB), Y-maze (working memory), novel object recognition, visual cliff (vision), sociability (social preference, i.e., other mouse over object), buried food finding (olfaction), sucrose preference (anhedonia), hot plate test (pain sensation), and hole board (working and reference memory). Moreover, automated home cage behavior analysis (LABORAS™), ultrasound vocalization recording, nest building (social competence), social tube (dominance), resident–intruder paradigm (aggression) and seizure propensity (seizure induction by pentylenetetrazole) were performed as indicated (Table 1a,b). In the following, the here relevant tests (with significant results) will be described in detail, while for the other tests the reader is politely referred to previous publications of ourselves and others (Kuc *et al*, 2006; Jamain *et al*, 2008; Mandillo *et al*, 2008; El-Kordi *et al*, 2012; Bodda *et al*, 2013).

### Automated home cage behavior analysis

Automated home cage behavior analysis was performed using LABORAS™ system (Metris b.v., Hoofddorp, the Netherlands), which consists of a triangular shaped sensor platform (Carbon Fiber Plate 1000 mm × 700 mm × 30 mm), positioned on 2 orthogonally placed force transducers (Single Point Load Cells) and a third fixed point attached to a heavy bottom plate (Corian Plate 980 mm × 695 mm × 48 mm). The whole structure stands on 3 spikes, which are adjustable in height and absorb external vibrations. Mice are housed in clear polycarbonate cages (Makrolon type II cage, 22 cm × 16 cm × 14 cm) with wood-chip bedding covered floors. The cage is placed directly onto the sensing platform, with the upper part of the cage (including top, food hopper and drinking bottle) suspended in a height-adjustable frame separate from the sensing platform. Resultant electrical signals caused by mechanical vibrations as induced by movement of the mouse are transformed by each force transducer, amplified to a fixed signal range, filtered to eliminate noise, digitized and stored on a computer. Stored signals are classified into separate behavioral categories like locomotor activity, immobility and climbing, and quantified by the LABORAS™ software. Prior to each session, LABORAS™ was calibrated. Spontaneous mouse behavior was assessed from 6:00 pm until 9:00am, with 1-h cage habituation prior to initiation of recording. Male (FVB/N: 10TG, 10WT; C57BL/6N: 12TG, 24WT) and female mice (FVB/N: 10TG, 10WT; C57BL/6N: 13TG, 28WT) were tested.

### Ultrasound vocalizations (USVs)

Ultrasound vocalizations (USVs) were recorded using a microphone (UltraSoundGateCM16) connected to a preamplifier (UltraSoundGate116), which was linked to a computer. At the day of recording, mice in their home cage (single-housed) were placed in the recording room for 60s. Subsequently, the intruder mouse was put into the resident's cage, and vocalization behavior recorded for 3 min. The intruder mouse was an anesthetized unfamiliar female (anesthetic: intraperitoneal injection of 0.25% tribromoethanol, 0.1 ml/10 g body weight). Number of calls per recording session was counted, and USVs were separated from other sounds using the whistles detection algorithm of Avisoft-SASLab 5.2 with following selection criteria: Possible changes per step = 4 (4687 Hz), minimal continuity = 8 ms, possible frequency range = 35–150 kHz. These criteria had been tested in former studies of mouse USVs (El-Kordi *et al*, 2012; Hammerschmidt *et al*, 2012). Avisoft Bioacoustics, Berlin, Germany, delivered all sound recording hardware and software. Male C57BL/6N mice (12TG and 23WT) were tested.

### The resident–intruder test

The resident–intruder test was used to study inter-male aggression in various independent cohorts of TG and WT mice of both genetic backgrounds (FVB/N and C57BL/6N) and different age groups, ranging from 8 to 32 weeks (Jamain *et al*, 2008; Bodda *et al*, 2013). As standard opponents (intruders), group-housed males (4 weeks younger than resident test males) of C57BL/6N background were employed (Charles River, Sulzfeld, Germany). An intruder was introduced into the home cage of the test resident. Observation started when the resident first sniffed the opponent and stopped (stop watch) at first attack (defined as bite) to prevent wounding, but lasted for 300s (FVB/N) or 600s (C57BL/6N) if no attack occurred (cut-off) (Mineur & Crusio, 2002; Pugh *et al*, 2004). Male mice (FVB/N: 12TG, 22WT; C57BL/6N: 10TG, 13WT) were tested. Over the first 180s (unless mice attacked before), frequency and duration of following behaviors was additionally quantified in C57BL/6N (10TG, 11WT): nose/snout and anogenital sniff, following/chasing.

### The social tube test

The social tube test measures social dominance (Messeri *et al*, 1975; Moretti *et al*, 2005; Garfield *et al*, 2011). The test apparatus comprises a 30-cm-long transparent acrylic tube with an internal diameter of 3 cm. Two mice are placed from opposite ends in the tube and gently pushed to the middle, where they face each other closely. Single-housed TG and WT C57BL/6N mice were challenged with unrelated group-housed mice of C57BL/6N background. A subject was declared “winner” when its opponent completely retreated from the tube (“out”) within 300s (cut-off). To account for both, winning/losing and time to win/lose, dominance values are calculated using the following formulas: winner dominance value = 100/time to out and loser dominance value = 1/(300 − time to out). Male C57BL/6N mice (10TG, 16WT) were tested in this paradigm.

### Nest building

Nest building is an important behavior in rodents, reflecting social competence in reproduction (Deacon, 2006; Satoh *et al*, 2011; El-Kordi *et al*, 2012). Two hours before dark phase, the nesting towel was removed from home cages of single-housed mice and replaced by a nestlet (pressed 2.7 g cotton square). Nest building quality was scored in the morning (Deacon, 2006; El-Kordi *et al*, 2012). Male C57BL/6N mice (10TG, 16WT) were tested.

### The pentylenetetrazole-induced seizure protocol

The pentylenetetrazole-induced seizure protocol has been described in detail previously (Ferraro *et al*, 1999; Bodda *et al*, 2013; Wojcik *et al*, 2013). Seizure activity was induced in wakeful mice by a single intraperitoneal (i.p.) injection of pentylenetetrazole (PTZ) (50 mg/kg body weight) followed by close observation for 30 min in a small, clear homecage. Latencies to focal (partial clonic), generalized clonic and maximal tonic-clonic behavioral seizures were recorded. Furthermore, 4 phases in the continuum of behavioral response to PTZ injection were defined as follows: (1) Hypoactivity (progressive decrease in motor activity until resting in a crouched or prone position with abdomen in full contact with cage bottom); (2) Partial clonus (clonus seizure activity affecting face, head, and/or forelimb or forelimbs); (3) Generalized clonus (sudden loss of upright posture, whole body clonus involving all 4 limbs and tail, rearing and autonomic signs); and (4) Tonic-clonic (maximal) seizure (generalized seizure characterized by tonic hindlimb extension—also associated with death). Finally, latencies to partial clonus (PC), generalized clonus (GC), and tonic-clonic (TC) seizures were summed to assign each mouse a seizure score that was used as a quantitative trait measure for mapping according to the following equation: Seizure score = (0.2)(1/PC latency) + (0.3)(1/GC latency) + (0.5)(1/TC latency)] × 1000. The weighting factors (0.2, 0.3 and 0.5) in the equation were included as means of incorporating a measure of the progressive nature of the PTZ-induced seizure phenotype into the severity rating because generalized clonus is regarded as a more significant event than partial clonus, and tonic hind limb extension is regarded as the most severe component of the phenotype. Therefore, the seizure score reflects the degree of progression of the seizure phenotype in each mouse.(Ferraro *et al*, 1999) The test was performed on adult *Mecp2* TG of FVB/N and C57BL/6N background (30–40 weeks old). Male (FVB/N: 6 TG, 10 WT; C57BL/6N: 12 TG, 10 WT) and female mice (FVB/N: 14 TG, 14 WT; C57BL/6N: 10 TG, 11 WT) were tested.

### Human sample

#### Schizophrenic patients (discovery sample)

The GRAS (Göttingen Research Association for Schizophrenia) data collection(Ribbe *et al*, 2010) was approved by the ethics committee of the Georg-August-University Göttingen (master committee) and respective review boards of collaborating centers. The project complies with the Helsinki Declaration. Patients fulfilling DSM-IV criteria for schizophrenia or schizoaffective disorder were included regardless of disease stage (acute, chronic, residual, or remitted). All study participants (European Caucasian 95.3%; other 2.0%; unknown 2.7%) and, if applicable, their legal representatives gave written informed consent. Of the included 1052 patients, 68.2% were male (*N* = 717) and 31.8% female (*N* = 335). Average age was 39.14 ± 12.56 years (range 17–78).

#### Healthy controls

Healthy voluntary blood donors were recruited by the Department of Transfusion Medicine at the Georg-August-University of Göttingen according to national guidelines for blood donation. As such, they widely fulfill health criteria, ensured by a broad predonation screening process including standardized health questionnaires, interviews, and assessment of hemoglobin concentration, blood pressure, pulse, and body temperature. Of the *N* = 1248 successfully genotyped control subjects (European Caucasian 97.8%; other 2%; unknown 0.2%), 61.5% were male (*N* = 768) and 38.5% female (*N* = 480). Average age was 37.44 ± 13.23 years (range 18–69).

#### Independent schizophrenia sample (replicate sample)

To replicate in an independent sample the phenotype–genotype associations found in male schizophrenic GRAS patients, data from male schizophrenic subjects (*N* = 385) of the Munich/Halle collection of Dan Rujescu could be analyzed (Van den Oord *et al*, 2006). Also in this replicate sample, written informed consent had been obtained from all subjects after detailed and extensive description of the study, which was approved by the local ethics committee and carried out in accordance with the ethical standards laid down in the Declarations of Helsinki.

#### Phenotyping—target variables

All schizophrenic patients of the GRAS data collection were comprehensively phenotyped (Ribbe *et al*, 2010). To prove our hypothesis that *MECP2* genotypes modulate aggressive behavior in human subjects, we selected target variables closely related to (*poor impulse control*) or predicting (*excitement*) aggressive behavior in schizophrenic individuals (Arango *et al*, 1999; Soyka *et al*, 2007; Colasanti *et al*, 2010). To assess the severity of *poor impulse control,* item 14 of the positive and negative syndrome scale (PANSS) (Kay *et al*, 1987) was used (“disordered regulation and control of action on inner urges, resulting in sudden, unmodulated, arbitrary or misdirected discharge of tension and emotions without concern about consequences”). From the subscale covering positive symptomatology, item 4 assessing excitement (“hyperactivity as reflected in accelerated motor behavior, heightened responsivity to stimuli, hypervigilance or excessive mood lability”) was employed (Tables 2 and 3). Both PANSS readouts were also available in the independent sample of schizophrenic individuals (replicate sample). The choice of items is supported by the literature: Cheung and colleagues compared aggressive and non-aggressive schizophrenia patients (aggression assessed by the Staff Observation Aggression Scale) with respect to single items of the PANSS (Cheung *et al*, 1997). The largest group difference was found for the PANSS item “poor impulse control”. Strikingly, the aggressive group had an average score of 4 (range: 1–7) on this item. Even after controlling for the total level of psychopathology, the associations of “poor impulse control” and aggressive behavior remained significant. Additionally, in a more recent prospective study, “poor impulse control” as measured by PANSS was highly predictive of aggressive behavior (assessed by the Overt Aggression Scale)(Nolan *et al*, 2005).

#### Phenotyping—control variables

Control variables (and potential confounders) are also presented in Tables 2 and 3. Sociodemographic data (age, years of education, unemployment rate), a cognition composite score and clinical variables describing disease severity were used to characterize the GRAS sample and exclude potential confounding effects explaining the target phenotype–genotype associations. The cognition composite score(Begemann *et al*, 2011) represents the mean of 3 z-standardized neuropsychological measures of higher cognitive functioning: reasoning ability (Leistungsprüfsystem subtest 3; Horn, 1983), executive functioning (Trail-Making Test B; Reitan, 1958) and verbal learning and memory (Verbal Learning and Memory Test; Helmstaedter *et al*, 2001). As further clinical variables, the general, positive and negative scores of the PANSS (target variables excluded from respective scores), chlorpromazine equivalents (standardized dosage of antipsychotic medication) (Rijcken *et al*, 2003; Woods, 2003) and Global assessment of functioning (GAF; Wittchen *et al*, 1997) as measure of impaired psychological, social and occupational performance were used.

#### DNA extraction, normalization, and genotyping

Genomic DNA was purified from whole blood using JETQUICK Blood & Cell Culture DNA Spin Kit (Genomed GmbH, Löhne, Germany) according to the manufacturer's protocol. Resulting DNA samples were aliquoted and stored at −80°C. For further analysis, DNA was normalized to 50 ng/μl with an automated robotic platform (Microlab Star, Hamilton, Bonaduz, Switzerland). For quality control, each sample was analyzed with a 0.8% agarose gel. Genotyping was performed using SimpleProbes (TIB Molbiol, Berlin, Germany) on LightCycler 480 (Roche Diagnostics, Basel, Switzerland), according to the manufacturer's instructions.

### Transfection studies

#### Cell lines

Human embryonic kidney 293 (HEK293) and mouse neuroblastoma (N2a) cells were maintained in DMEM supplemented with 1 g/l glucose, L-glutamine (Glutamax), 5% fetal calf serum, 100U/ml penicillinG sodium and 100 μg/ml streptomycin sulfate. For luciferase assays, cells were seeded into 12-well plates (220,000 cells/well/2 ml medium), cultured for 24 h and co-transfected using Lipofectamine 2000 (Invitrogen, Karlsruhe, Germany), following the manufacturer's instructions.

#### Luciferase reporter constructs and detection assay

The reporter plasmids phRL-TK rs2734647C, or rs2734647T, respectively, were constructed by cloning a 3′UTR fragment of 346 bp and including the SNP downstream of the Renilla luciferase open reading frame, making use of the XbaI restriction site of phRL-TK (forward primer: 5′-ATTATCTAGACCAGGTCTACCCCTCCCGGC-3′, reverse primer: 5′-ATTATCTAGAGGCTGCTCCCTGTCCCAGGT-3′). Sequence integrity was verified using Sanger sequencing. Of the Renilla luciferase reporter construct (phRL-TK rs2734647C, or rs2734647T, respectively), 1 μg (per well), plus 1 μg (per well) of the reference construct pCMV-LacZ (Clontech, Mountain View, CA, USA) were co-transfected in the presence of 10 pg of mirVana miRNA mimic hsa-miR-4711-3p, hsa-miR-511, hsa-miR-515-3p, hsa-miR-519e-3p, negative control #2 (all Life Technologies, Darmstadt, Germany), or no miRNA, respectively. After 24 h, cells were split in 96-well plates, creating four technical replicates for each condition, and separately for luciferase and beta-galactosidase measurement. Enzyme activity was determined using a Mithras LB 940 Plate Reader (Berthold, Bad Wildbad, Germany). Renilla luciferase activity was normalized to beta-galactosidase activity.

### RNA isolation and quantitative PCR

Peripheral blood mononuclear cells (PBMC) were isolated from citrate blood of men with rs2734647C or rs2734647T genotype (X-chromosomal gene), applying a standard isolation procedure (Ficoll-Paque Plus, GEHealthcare, München, Germany). Human RNA was extracted from deep-frozen human brain samples of adult male subjects who had been free of neuropsychiatric diseases, from placenta or PBMC, respectively, using a miRNeasy Mini kit (Qiagen, Hilden, Germany). The same kit was employed for isolation of mouse RNA from whole E17 embryo (divided into body and head), hippocampus, amygdala, placenta, and cultured microglia. Synthesis of cDNA was done by the SuperScriptIII system (Invitrogen, Karlsruhe, Germany). Detection of *MECP2* cDNA was performed using SYBR green (Roche, Diagnostics GmbH, Mannheim, Germany) and specific primer pairs amplifying the *MECP2*_e2 isoform (NCBI reference sequence NM_004992), spanning exons 2–3 (forward primer: 5′- CAGCTCCAACAGGATTCCAT-3′, reverse primer: 5′- TGGAGGTCCTGGTCTTCTGA-3′), or both isoforms (NM_004992 and NM_001110792), spanning exons 3–4 (forward primer: 5′- AGCTTAAGCAAAGGAAATCTGG-3′, reverse primer: 5′-GCTTTTCCCTGGGGATTG-3′). Specific Taqman microRNA assays were used to detect hsa-miR-511, hsa-miR-4711-3p, or mmu-miR-511 (Applied Biosystems, Foster City, CA, USA), following the manufacturer's instructions. *MECP2* expression levels were normalized to *GAPDH*, human miRNA expression levels to RNU43, and mouse miRNA expression levels to sno-142.

### Statistics

All experimental data acquisition was done by experimenters unaware of group assignment (“‘blinded”). Mouse behavioral data were analyzed by Mann–Whitney *U*-test or 2-way analysis of variance including *post hoc* Bonferroni testing, where applicable, using Prism4 (GraphPad Software, San Diego, CA, USA). All data are presented as mean ± s.e.m., unless stated otherwise. Luciferase assay results from each experiment were normalized to the relative luciferase activity without miRNA co-transfection and analyzed using one-tailed unpaired *t*-tests. qPCR results were normalized to the respective control genes and analyzed using one-tailed unpaired *t*-tests. PLINK (v1.07) (Purcell *et al*, 2007) was used for the analysis of statistical association between single SNPs and case or control status (allelic test), and to test for deviations from Hardy–Weinberg equilibrium. Statistical analyses of phenotype–genotype associations in the human samples (both GRAS and replicate sample) were performed using SPSS for Windows version 17.0 (SPSS Inc., Chicago, IL, USA; http://www.spss.com). As *MECP2* is X-linked, analyses were performed separately for men and women. Genotype differences with respect to the target variables were assessed by analysis of covariance. Covariates age at examination, years of education, chlorpromazine equivalents and severity of negative symptoms (PANSS) were used, as these parameters are likely to influence the extent to which impulsive aggressive behavior becomes obvious in a social situation. For the cognition composite score, analysis of covariance with covariates age, chlorpromazine equivalents and severity of negative symptoms was applied as these variables have been widely reported to confound performance on neuropsychological tests(Bilder *et al*, 1992; Hori *et al*, 2006). Genotype differences with respect to sociodemographic and clinical measures were tested non-parametrically using chi-square (nominal variables) or Mann–Whitney *U*/Kruskal–Wallis tests (continuous variables). All *P*-values derived from statistical models for the GRAS sample are two-sided (Tables 2 and 3). For the replicate sample one-sided *P*-values are displayed (Table 4). Nominal significance level for all analyses was set to *P < 0.05*.

## The paper explained

### Problem

The transcriptional regulator methyl-CpG-binding protein2, MECP2, is renowned because of the devastating neurodevelopmental disorder Rett syndrome, caused by partial or complete loss of its function. The very same gene, when duplicated, induces a similar disorder, indicating the necessity of tight *MECP2* regulation. Among the vast array of other genes influenced by MECP2, many are involved in modulating behavior. Surprisingly, nothing is known on the physiological (i.e., non-disease-related) effects on behavior of subtle *MECP2* expression differences.

### Results

We present here a translational study that explores behavioral consequences of mildly increased *MECP2* expression across species. We find that the behavioral target domain directed by MECP2 is male social aggression. This in turn is subject to modification by the genetic background, as we demonstrate by comparing an inherent aggressive with a less aggressive mouse strain. In two independent cohorts of schizophrenic individuals,that is, a discovery and a replicate sample, we identify a role of *MECP2* in aggressive human behavior and show that a polymorphism in the untranslated region of the gene determines binding efficiency of another brain-expressed regulator, microRNA-511. Notably, the genotype-dependent expression difference of 40–50% (rs2734647-C>T) in man, at least partially mediated by microRNA-511, is comparable to the level of transgenic overexpression in our mice, emphasizing the physiological significance of these findings.

### Impact

Genetic regulation of behavior is still poorly understood. We explore for the first time behavioral consequences of mildly (∼50%) increased *MECP2* expression in mouse and man. This is achieved by transgenic *Mecp2* overexpression in mice and—in parallel—through genetic variation-induced non-regulability of *MECP2* gene expression (rs 2734647-C: no miR-511-mediated *MECP2* downregulation) in humans. We find MECP2 to be a modulator of social aggression. While the mouse data shows an interaction between genetic background and *Mecp2* expression on behavior, the human data provides means by which genetic variation may affect *MECP2* expression and result in behavioral change.
