# Supplementary material for: Mild expression differences of MECP2 influencing aggressive social behavior
Source: EMBO Mol Med. 2014 Mar 20;6(5):662–84. doi: 10.1002/emmm.201303744 (PMC4023888; doi:10.1002/emmm.201303744)
Supplement: Supplementary file 1 [file emmm0006-0662-sd1.pdf]

Mild expression differences of MECP2 influencing aggressive social behavior

Martesa Tantra, Christian Hammer, Anne Kästner, Liane Dahm, Martin Begemann, Chiranjeevi Bodda, Kurt Hammerschmidt, Ina Giegling, Beata Stepniak, Aracely Castillo Venzor, Bettina Konte, Begun Erbaba, Annette Hartmann, Asieh Tarami, Walter Schulz-Schaeffer, Dan Rujescu, Ashraf U. Mannan, and Hannelore Ehrenreich

Corresponding author: Hannelore Ehrenreich, Max Planck Institute of Experimental Medicine

Review timeline:

Submission date:	26 September 2013
Editorial Decision:	05 November 2013
Resubmission:	04 December 2013
Editorial Decision:	18 January 2014
Revision received:	23 January 2014
Accepted:	27 January 2014

Transaction Report:

(Note: With the exception of the correction of typographical or spelling errors that could be a source of ambiguity, letters and reports are not edited. The original formatting of letters and referee reports may not be reflected in this compilation.)

Editor: Céline Carret

1st Editorial Decision 05 November 2013

Thank you for the submission of your research manuscript to our editorial office. We have now received the enclosed reports from the three referees who were asked to review it.

As you will see, all referees find the topic of interest and referee 2 is rather positive about it. However, overall, all referees share the same concerns about the limited behavioural studies and mechanistic insights. All of reviewers call for a considerable amount of additional experimentation to resolve these issues, some of which would likely require a lot of time.

Given the nature of these criticisms, the amount of work likely to be required to address them, and the fact that EMBO Molecular Medicine can only invite revision of papers that receive enthusiastic support from a majority of referees, I am afraid that we do not feel it would be productive to call for a revised version of your manuscript at this stage and therefore we cannot offer to publish it.

Nevertheless, the three referees clearly find the study of interest, and as we also do, we would have no objection to consider a new manuscript on the same topic if at some time in the near future you obtained data that would considerably strengthen the message of the study and address the referees concerns in full. To be completely clear, however, I would like to stress that if you were to send a new manuscript this would be treated as a new submission rather than a revision and would be reviewed afresh, in particular with respect to the literature and the novelty of your findings at the

time of resubmission. If you decide to follow this route, please make sure you nevertheless upload a letter of response to the referees' comments.

At this stage of analysis, though, I am sorry to have to disappoint you. I nevertheless hope, that the referee comments will be helpful in your continued work in this area and I thank you for considering EMBO Molecular Medicine.

***** Reviewer's comments *****

Referee #1 (Comments on Novelty/Model System):

The study is done in a very superficial manner. there is no depth to behavioral studies (over time) to permit an unequivocal conclusion and the molecular data on the miRNA are weak.

Referee #1 (Remarks):

The manuscript by Tantra et al., is interesting in that it explores the consequences of mild changes in MeCP2 expression and extend the study to potential impact of such variations in humans. There are two components to this study. The first pertains to consequences of mild over-expression (50%) on mouse phenotypes. The authors show increased aggression in FVB background, but not in the C57BL6 background. In fact, aggression is reduced in the latter background. They also uncover increased susceptibility to seizures. The findings are interesting, but they could have been extended further because as is they have limitations.

1) Behavioral assays started at ~5 weeks and most revealed no phenotypes. Thus, the authors can't really make conclusions about mild Mecp2 over-expression and such phenotypes as it might take time to uncover phenotypes. Ideally detailed behavioral analysis beyond 20-25 weeks would be the only way to know whether mild over-expression can or cannot cause various phenotypes.

2) The difference in aggressive behavior due to genetic background is interesting, but the authors do not attempt any experiments (in the context of their findings to explain that difference). For example, do levels of miRNA-511 vary in a region-specific manner in the two strains? Do levels of MeCP2 vary in different brain regions in the two strains? It is conceivable that other factors modulate the phenotypes, but addressing the above questions is very basic in the context of this study.

The human association data and the uncovering of the variant at rs2734647 are interesting. However, here again there are a few issues that must be addressed.

1) The human data reveal increased aggression in face of decreased MeCP2, whereas the mouse data focus on over-expression. This requires some deeper discussion.

2) The authors need to provide additional molecular evidence about the relationship between miRNA-511 and MeCP2. The way I understand this miRNA has no effect on MeCP2 unless the 3' UTR of MeCP2 has a "T" in the miRNA target site. This means the majority of human alleles (CC) cannot be targeted by this miRNA. If this is the case then the whole argument about the functional relevance of this discovery and miRNA regulation of MeCP2 by this miR-511 is biologically questionable. This would argue that this miRNA only regulates MeCP2 in the "T" carriers. To be convincing, the authors should consider other targets of miRNA-511 and explore if such variations in the target sequence also occur.

Incidentally, only the C allele is conserved with mouse 3'-UTR, which also has a C. But the mouse and human miRNAs have differences in the seed sequence such that the human miRNA only can regulate the "T" allele in MeCP2. This is very unusual and far away from what one would predict if

this was evolutionarily and biologically relevant.

Referee #2 (Comments on Novelty/Model System):

This is a very interesting study associating aggressive social behavior with MECP2 genotype, genetic background and expression changes.
Some issues need to be clarified but it has enough merits for publication in EMBO MM.

Referee #2 (Remarks):

A brief description of the transgenic models should be included. The authors cant assume that the readers are familiar with their published work. They have to clarify that this is BAC transgene to let the readers know that it use the endogenous promoter, if both Mecp2 informs are expressed and whether the GFP tag was fused at the N or C terminus.

Provide a possible explanation for the sexual dimorphism in spontaneous locomotor activity (unchanged for males of both strains).

The phrase: "..FB/N mice exposed to inferior and per se less aggressive intruders.." does not make sense, What is the meaning of "inferior" in this context?

The resident intruder test was not performed in the same conditions in both compared strains (preheating was used for the C57 mice and not for the FVB mice), and thus it is not clear that the difference will persist were both in tested in similar conditions. Most probably yes, but it needs to be shown.

In addition, the effect of the preheating is a confounding factor, the authors should exclude a differential sensitivity to heat of the C57 TG vs c57 WT, a factor that could strongly influence their aggressive behavior, as stated by the authors. In other words if the TG have decreased sensitivity to heat (Rett patients have altered pain sensitivity) the heat threshold to increase the level of aggression could be elevated and the observed differences in response attributed exclusively to sub-threshold induction of aggression in TG vs. induction in WT. The tube test is an indirect measure of aggressiveness, the lack of significance in this test (although there is a trend toward a differential response) highlights the need for a test of heat sensitivity to substantiate their conclusion.

Inferiority was used again as representing a diminished response vs the wild type, which I believe is not an appropriate use of the term.

Please note if males and females were used in the tests depicted in fig 2 and 3. This is important in the light of sex-dependent responses in sociability and startling and the male specific association of MECP2 genotypes with aggression readouts. In addition, the hypoactivity seen in the TG females could complicate the analysis of the tests.

The authors need to clarify that the association studies with aggression and impassivity were performed on a population of schizophrenia cases and not a combined case-control population or a pure control popyulation. The replication cohort is reported as schizophrenic, raising the possibility of a genetic (or phenotypic) interaction. It would be important to screen a non-schizophrenic population differing for aggressive traits to test if the association still holds true.

Please note which is the major allele for rs2734647 and rs 2239464.

The levels of expression of the putative miRs in HEK and N2A cells should be measured, as the results could be affected by the endogenous amounts of these.

What are the reported values for miR-511 expression in mouse tissues?

© EMBO

The N in figure 6F refers to carrier individuals or experimental replicates?

The perfect match of the mouse miR-511 with 3'UTR C allele vs the human miR-511 matching the T allele is a very pertinent observation. It will be interesting to determine whether the FVB and C57 strains are polymorphic for the Mecp2 UTR allele, or the miR-511 sequence.

Minor details:

Abstract: (Typo) In the first line of the abstract, the number 2 in methyl-CpG-binding protein2 should be separated by a space.

Introduction: Most of Rett causing mutations are not Loss-of-function alleles as suggested in the first paragraph.

The phrase "Strikingly, females exhibited mild psychiatric symptoms despite 100% skewing of inactivation of the mutated allele and normal MECP2 mRNA levels (Ramocki et al, 2009)" seems out of context. It is unclear what is the point thee authors are trying to make.

The relationship of the SNP rs2734647 with the SNP (c.*3638A>G)should be explained for non-genetic experts.

Figures: Fig 4:The schematic representations of Mecp2 isoforms is confusing. These are splicing isoforms and the existence of them is dependent on intron elimination; thus depiction of introns is incorrect. The genomic organization of the locus should be presented with the location of the considered SNPS.

Fig 6A: the meaning of the numbers surrounding the miRNA sequence should be clarified. Fig 6B and C: note which baseline was used for statistical significance determinations (negative control or the alternative allele?)

Referee #3 (Comments on Novelty/Model System):

The authors present human data indicating an association between decreased MECP2 expression, SNP, and clinical features, whereas they present animal data showing mild overexpression of MECP2 leading to a phenotype. They do not discuss this difference adequately.

Referee #3 (Remarks):

The authors provide additional characterization of a mouse with a 50% overexpression of MeCP2 protein. They identify an increased susceptibility to seizures, decreased locomotor activity, strain dependent modification of aggressive behavior, disrupted social behaviors, and enhanced acoustic startle response. The authors also identify polymorphisms in MECP2 that correlate with increased surrogate measures of aggressive behavior in human male schizophrenia patients. Additionally the authors propose and test a hypothesis regarding microRNA regulation of MECP2 as the mechanism contributing to the altered aggression measures.
A relationship between MeCP2 function and aggressive behavior, social interactions, and motor function have been demonstrated in literature cited by the authors. Furthermore, 50% changes in MeCP2 expression have been demonstrated to cause behavioral abnormalities in mice in a 2008 HMG paper by Samaco et al (which the authors should cite) and in the Kerr paper already cited by the authors. The major novelty of the paper is in the identification of MECP2 polymorphisms that modify aggressive behavior in humans, and the identification of the interaction between miR-511 and the 3'UTR of MECP2 as the effector of this phenotype.

The human findings merit publication, however several concerns regarding methods and interpretations of the data should be addressed.

Concerns:

1. The authors modified the protocol used to test aggressive behavior in the C57 mouse strain by placing the mice in a warmer environment before and during the test. However, no specific precedent is given/cited for why those changes would be expected to increase the aggressive behavior to detectable levels in the mice. Citation or empirical evidence should be added.

2. Control social behavior tests involving follow/chase, sniffing, nest building etc. are only presented for the C57 strain and are used to provide support for the difference in aggressive behavior between the strains. However, the diminished activity in the C57 strain could also be an explanation. Similar social behavior tests should be presented for the FVB strain for a more effective argument regarding strain (genetic background) effects on social behavior.

3. The inclusion of the female mouse data is appreciated. However, because the transgene is not confirmed to be localized to the X chromosome, translational relevance is diminished by lacking the ability to model mosaicism from XCI.

4. The language should be tempered when using the mouse results for rationale of the human investigation. The stated mouse data indicates increased MeCP2 expression may increase or decrease aggressive behavior in a strain dependent manner, but the authors insist on only using the increased aggression from the FVB strain as a justification to look for increased aggressive behavior in people. Humans are not an isogenic population, the authors fail to address the prediction of a population with reduced aggression suggested by the C57 mouse strain data (i.e. a bimodal aggression distribution when combining the different backgrounds).

5. Although the authors choice of the PANSS to indirectly assess aggressive behavior is supported in the literature, their selection of only 2 individual subscores appears excessively reductive. Empirical evidence or citation of these specific subscores as predictive of aggressive behavior would be advised.

6. The language in the abstract and Impact sections should be tempered regarding interactions between MECP2 expression and genetic background. While the mouse data provides evidence of an interaction between genetic background and MeCP2 expression on behavior. The human data only provides evidence of a means by which genetic background can effect MeCP2 expression and result in behavior change.

7. The authors should be aware that the rs2734647 variant has been associated with increased risk of schizophrenia in a Han Chinese population (Wong et al. Schizophr Bull. 2013), since they claim it does not increase risk in the European Caucasion population used in their study.

8. Given that the ancestral and predominant allele at the locus of rs2734647 is C and the T allele shows reduced expression, the hypomorph Mecp2tm1Bird mouse (Kerr 2008, Samaco 2008) may be a better model of the human MECP2 expression difference observed in this paper. This is one of the biggest problems with this paper-the animal work is based on mild overexpression whereas the human condition mentioned is based on mild decreased expression. The authors cannot claim a direct relationship between these two-at best they can make the claim that EITHER mild over or under expression can lead to a phenotype, either in mice (under and over) or people (only evidence in people).

© EMBO

Resubmission 04 December 2013

Thank you very much for having our manuscript "Mild expression differences of MECP2 influencing aggressive social behavior" reviewed by expert referees of EMBO Molecular Medicine.

As you will see in our point-to-point response, the concerns raised by the reviewers and summarized in your decision letter ('limited behavioural studies and mechanistic insights'), are essentially based on misunderstandings. Therefore, we decided to resubmit the paper now, after careful revision (changes marked in yellow), addition of a large amount of new data, and clarification of all points of concern.

Specifically, we have added a new table which includes all the comprehensive behavioral studies that have been performed in several independent cohorts of both mouse strains. In the previous version of the manuscript they had only been mentioned, but not presented, due to the essentially negative results. We hope that this will convince you that the behavioral studies were by no means 'limited or superficial'.

Moreover, we have clarified the major point of conceptual misunderstanding: The direction of altered MECP2 gene expression that we find associated with altered aggression in mice and man is the same, i.e. an increase (~50%). In the former, this increase is 'artificially' achieved by transgenic overexpression, whereas in the latter, it results from a lack of miR-511 induced downregulation, due to the base exchange (C versus T) in the 3'UTR SNP rs2734647, positioned in the seed region of miR-511. See also our sketch in the point-by-point response. This has now been emphasized in several positions in the paper including abstract and impact section.

In addition, we have added new data on expression of miR-511 in the brains of both mouse strains, C57BL/6N and FVB/N (even though we feel that this is not really relevant for the message of the present paper). Also, we have addressed point-by-point all other questions or concerns that the reviewers had, and integrated their suggestions in the paper wherever possible.

We hope that you will realize that most points were based on totally wrong assumptions, and will accept our response and the revised version of the paper which we sincerely hope is now suitable for publication in EMBO Molecular Medicine. We are looking forward to your response.

© EMBO

Point-by-point response to the reviewers' comments

In the following, the reviewers' comments are presented in Courier New, font 10, and our point-by-point response is given in Arial, font 11, bold.

Referee #1 (Comments on Novelty/Model System):

The study is done in a very superficial manner. there is no depth to behavioral studies (over time) to permit an unequivocal conclusion and the molecular data on the miRNA are weak.

We are sorry that the reviewer became the impression that the behavioral studies were done in a superficial manner. This impression must have come from us presenting only a small part of all the extensive behavioral work that we have performed. In fact, we had mentioned most of this work as 'data not shown' in the previous version of the manuscript since we felt that the presentation of large amounts of negative data would not add to the paper. Driven by the reviewer's totally wrong impression, we have now prepared a large table (new Table1a and b) that gives a summary of the most important tests performed in several independent cohorts of mice of the 2 strains, of both genders and of various age groups.

Moreover, we have now clarified the misunderstandings regarding miR-511 which obviously led to the conclusion of 'weak data' (see points below).

Referee #1 (Remarks):

The manuscript by Tantra et al., is interesting in that it explores the consequences of mild changes in MeCP2 expression and extend the study to potential impact of such variations in humans. There are two components to this study. The first pertains to consequences of mild over-expression (50%) on mouse phenotypes. The authors show increased aggression in FVB background, but not in the C57BL6 background. In fact, aggression is reduced in the latter background. They also uncover increased susceptibility to seizures. The findings are interesting, but they could have been extended further because as is they have limitations.

We thank the reviewer for his/her nevertheless encouraging comments and the time he/she invested into reading and commenting on our manuscript. In the following, we have addressed the points of concern.

1) Behavioral assays started at ~5 weeks and most revealed no phenotypes. Thus, the authors can't really make conclusions about mild Mecp2 over-expression and such phenotypes as it might take time to uncover phenotypes. Ideally detailed behavioral analysis beyond 20-25 weeks would be the only way to know whether mild over-expression can or cannot cause various phenotypes.

We thank the reviewer for making us aware of the importance to present all our original data. We have now added a table (new Table1a and b) that shows that mice have been tested in various cohorts and different ages, with essentially consistent results.

2) The difference in aggressive behavior due to genetic background is interesting, but the authors do not attempt any experiments (in the context of their findings to explain that difference). For example, do levels of miRNA-511 vary in a region-specific manner in the two strains? Do levels of MeCP2 vary in different brain regions in the two strains? It is conceivable that other factors modulate the phenotypes, but addressing the above questions is very basic in the context of this study.

The questions raised by the reviewer are interesting, but not really relevant for the present paper. Our mouse model is based on mild transgenic overexpression of *Mecp2*, i.e. an 'artificial' situation that leads to strain-specific modulation of aggressive behavior.

In the translational part, we searched for physiological correlates of increased *MECP2* expression and altered aggression in the human sample. We found an association of defined *MECP2* genotypes with aggression readouts, and thus hypothesized that we might find – in analogy to the TG mice – mild *MECP2* 'overexpression' as an underlying mechanism of genotype differences.

Indeed, we identified miR-511 as one factor (out of certainly many others) regulating *MECP2* expression. The miR-511 seed sequence is located in the 3'UTR with the respective SNP (C/T) determining miR-511 binding probability. In case of the C allele, miR-511 responsive downregulation of gene expression is lacking, i.e. the C allele results in higher *MECP2* mRNA levels and higher aggression.

Therefore, for the message of the present paper, comparative measuring of miR-511 expression in the two *Mecp2* overexpressing mouse strains is not too helpful. In the mouse, no analogous SNP in the respective region of the gene is known (see also point below). The mouse miR-511 therefore is supposed to bind in any case. And we do not expect miR-511 to be the sole responsible factor for modulating basic aggression. Of course, it may well be that there is a mouse strain-dependent difference in the contribution of miR-511 expression to basal levels of aggression. But this has not been the topic of our work and would have had to be approached entirely differently.

We have now made this clearer in the manuscript (page 4, para 4 end of introduction). Nevertheless, we have added comparative data on strain specific brain expression of miR-511 into the paper (page 10, para 1; page 14, para 1).

The human association data and the uncovering of the variant at rs2734647 are interesting. However, here again there are a few issues that must be addressed.
1) The human data reveal increased aggression in face of decreased MeCP2, whereas the mouse data focus on over-expression. This requires some deeper discussion.

This is an unfortunate misunderstanding by the reviewer. The human data revealed increased aggression in case of non-regulability of MECP2 expression by miR-511 (rs 2734647 C carriers show no miR-511-mediated down-regulation), and therefore increased expression. This is consistent with the effect in FVB/N mice, i.e. higher aggression in case of TG *Mecp2* overexpression. See also sketch below.

2) The authors need to provide additional molecular evidence about the relationship between miRNA-511 and MeCP2. The way I understand this miRNA has no effect on MeCP2 unless the 3' UTR of MeCP2 has a "T" in the miRNA target site. This means the majority of human alleles (CC) cannot be targeted by this miRNA. If this is the case then the whole argument about the functional relevance of

this discovery and miRNA regulation of MeCP2 by this miR-511 is biologically questionable. This would argue that this miRNA only regulates MeCP2 in the "T" carriers.

The reviewer is right saying that our data show that miR-511 regulates MECP2 expression only in T carriers. Also, T is indeed the minor allele, i.e. occurs in >15% males and >30% females, and we do not have any evidence for more than one miR-511 binding site in the *MECP2* 3'UTR. However, we do not understand the reviewer's remark that this would be biologically questionable. Why?

To be convincing, the authors should consider other targets of miRNA-511 and explore if such variations in the target sequence also occur.

Demanding the investigation of putative other (certainly existing) miR-511 targets is not helpful to explain or upgrade the here documented and replicated association in men between aggression and the *MECP2* SNP rs2734647. We are not investigating the global role of miR-511 in regulating aggression. This would be an entirely other story. Importantly, we do not claim that the *MECP2* SNP alone is responsible for, or even allows prediction of an individual's aggressive behavior. We present and replicate a statistical association of a genetic *MECP2* variant with the complex phenotype 'aggression' in a large population, stressing the role of *MECP2* in aggression-related phenotypes - not more and not less.

Incidentally, only the C allele is conserved with mouse 3'-UTR, which also has a C. But the mouse and human miRNAs have differences in the seed sequence such that the human miRNA only can regulate the "T" allele in MeCP2. This is very unusual and far away from what one would predict if this was evolutionarily and biologically relevant.

Again, this is a somewhat surprising statement. Although we do not speculate about the exact nature of selective pressures leading to the constellation of miR-511 and rs2734647 in humans, it is feasible that positive selection has promoted the dissemination of the rs2734647 T allele in the human population, e.g. as a counter-reaction to a possible loss of miR-511 mediated regulability after a mutation in the miR-511 seed. Such unusual constellation is unlikely coincidental and the reason why it plausibly supports biological relevance.

Referee #2 (Comments on Novelty/Model System):

This is a very interesting study associating aggressive social behavior with MECP2 genotype, genetic background and expression changes. Some issues need to be clarified but it has enough merits for publication in EMBO MM.

We thank the reviewer for his positive and stimulating comments and the time he/she invested into reading and commenting on our manuscript. In the following, we have addressed all points of concern.

Referee #2 (Remarks):

A brief description of the transgenic models should be included. The authors cant assume that the readers are familiar with their published work. They have to clarify that this is BAC transgene to let the readers know that it use the endogenous promoter, if both Mecp2 informs are expressed and whether the GFP tag was fused at the N or C terminus.

We thank the reviewer for this constructive suggestion. A description of the generation of the transgenic models has now been included (page 14, para 3; page 15, para 1).

Provide a possible explanation for the sexual dimorphism in spontaneous locomotor activity (unchanged for males of both strains).

We thank the reviewer for this remark. Since the mentioned sexual dimorphism regarding spontaneous locomotor activity was only observed in FVB/N mice (not in C57BL6/N; Figure 1), we had not further commented on it in the paper. The interesting point 'sexual dimorphism', however, has now been added to the manuscript, since it is consistently found e.g. in the startle response (independent of the genetic background).

There have indeed been reports on sexual dimorphism with respect to *Mecp2*/Mecp2 expression and function in the brain. For instance, in amygdalae and ventromedial hypothalamus, male rats express less *Mecp2* as compared to females (Kurian et al. 2007). Furthermore, conditional knockout of *Mecp2* during amygdala development caused subtle modifications of juvenile play behavior in male but not female rats (Kurian et al. 2008). These findings indicate a role of Mecp2 in gender-specific modulation of behavior. This has now been added to the discussion (page 13, para 3).

The phrase: "..FB/N mice exposed to inferior and per se less aggressive intruders.." does not make sense, What is the meaning of "inferior" in this context?

'Inferior' is referring to the lower body weight of the younger intruders. In order to avoid misunderstandings, the phrase has now been changed as follows: "*(1)* We aimed at testing reproducibility of this phenomenon in FVB/N mice exposed to younger intruder males of C57BL6/N background, i.e. *per se* less aggressive intruders (Mineur & Crusio, 2002; Pugh et al, 2004)." See page 5, para 3.

The resident intruder test was not performed in the same conditions in both compared strains (preheating was used for the C57 mice and not for the FVB mice), and thus it is not clear that the difference will persist were both in tested in similar conditions. Most probably yes, but it needs to be shown.

As the reviewer will see, we had indeed performed the testing at first in similar conditions in both strains (see new Table1b). We did this test even in different age groups. However, when the protocol used for testing aggression in male FVB/N was applied to male C57BL6/N, not the slightest attack was registered over a 5min observation period (new Table 1b). Therefore, we modified the protocol for the male C57BL6/N by introducing a warmer temperature (a warming pad set to 38°C) and prolonged the observation period to 10min. This finally gave the reported aggression results.

The ambient temperature is known to influence aggression in mice (Greenberg, 1972; Gaskill et al., 2012). Importantly, the warming pad temperature (38°C) was far below the value that is used to measure pain sensitivity (for the hot plate test, the temperature was set to 55°C). This information has now been added to the manuscript, page 5, para 3; page 6, para 1.

In addition, the effect of the preheating is a confounding factor, the authors should exclude a differential sensitivity to heat of the C57 TG vs c57 WT, a factor that could strongly influence their aggressive behavior, as stated by the authors. In other words if the TG have decreased sensitivity to heat (Rett patients have altered pain sensitivity) the heat threshold to increase the level of aggression could be elevated and the observed differences in response attributed exclusively to sub-threshold induction of aggression in TG vs. induction in WT.

As displayed in the new Table1b, no genotype differences were detected with respect to pain sensitivity as measured by the hot plate test at 55°C.

The tube test is an indirect measure of aggressiveness, the lack of significance in this test (although there is a trend toward a differential response) highlights the need for a test of heat sensitivity to substantiate their conclusion.

This had already been performed (new Table1b). Please see also responses above.

Inferiority was used again as representing a diminished response vs the wild type, which I believe is not an appropriate use of the term. Please note if males and females were used in the tests depicted in fig 2 and 3. This is important in the light of sex-dependent responses in sociability and startling and the male specific association of MECP2 genotypes with aggression readouts. In addition, the hypoactivity seen in the TG females could complicate the analysis of the tests.

This seems to be a misunderstanding. In Figures 2 and 3, only data from male mice are presented (see figure legends). The hypoactivity seen in the TG females does not have any impact here.

But, to be clear, there is no hypoactivity in females nor in males upon testing in the open field (see new Table1). Only LABORAS, i.e. spontaneous home cage behavior shows less locomotion.

The authors need to clarify that the association studies with aggression and impassivity were performed on a population of schizophrenia cases and not a combined case-control population or a pure control population. The replication cohort is reported as schizophrenic, raising the possibility of a genetic (or phenotypic) interaction. It would be important to screen a non-schizophrenic population differing for aggressive traits to test if the association still holds true.

We agree with the reviewer that it would be interesting to have a healthy population as well. Unfortunately, the PANSS instrument is normally not done in healthy individuals, therefore an adequate sample would be hard to find. The use of other (non-cross validated) instruments for measuring aggression in turn would not be helpful for replication. And even here, adequately sized control populations are not trivial to find. Nevertheless, aggression is not a core symptom of schizophrenia, and association of this trait in 2 independent cohorts with *MECP2* genotypes – on top of the aggression association of mild *Mecp2* overexpression in mice – should be sufficiently convincing.

Please note which is the major allele for rs2734647 and rs 2239464.

This information has now been specifically added to the manuscript, page 8, para 2.

The levels of expression of the putative miRs in HEK and N2A cells should be measured, as the results could be affected by the endogenous amounts of these.

We thank the reviewer for his remark. Since we used adequate (negative, i.e. the non-miR-transfected) controls, the results of the Luciferase assay should not have been affected. Nevertheless, we have now performed a new miR-511 assay and have included this information in the paper, page 9, para 1.

What are the reported values for miR-511 expression in mouse tissues?

Unfortunately, we have not found a single publication reporting these values. This is why we analyzed for our own interest the levels in various mouse tissues (see manuscript page 10, para 1). This is, however, not really relevant for the main message of the present paper.

The N in figure 6F refers to carrier individuals or experimental replicates?

Sorry for not making this clearer in the first place. The N refers to individuals. This has now been added to the Figure6 legend.

The perfect match of the mouse miR-511 with 3'UTR C allele vs the human miR-511 matching the T allele is a very pertinent observation. It will be interesting to determine whether the FVB and C57 strains are polymorphic for the Mecp2 UTR allele, or the miR-511 sequence.

The two mouse lines are not polymorphic for the *Mecp2* 3'UTR allele or the miR-511 sequence. This was checked using data available from the Jackson Laboratories (http://www.jaxlab.org). We agree, however, that it would be interesting to determine if non-inbred mice are polymorphic for the respective alleles. This idea has now been added to the manuscript, page 10, para 4; page 11, para 1.

Minor details:

Abstract: (Typo) In the first line of the abstract, the number 2 in methyl-CpG-binding protein2 should be separated by a space.

This has now been done.

Introduction: Most of Rett causing mutations are not Loss-of-function alleles as suggested in the first paragraph.

The reviewer may refer to e.g. hypomorphic alleles resulting in truncated forms of the gene that retain partial function. Therefore, to be very clear in the manuscript, we have now modified the sentence to say: 'Complete or partial loss-of-function mutations of *MECP2* lead to Rett …' Page 3, para 1; page 25, para 1.

The phrase "Strikingly, females exhibited mild psychiatric symptoms despite 100% skewing of inactivation of the mutated allele and normal MECP2 mRNA levels (Ramocki et al, 2009)" seems out of context. It is unclear what is the point thee authors are trying to make.

The reviewer is right. The citation does not fit perfectly in this context. We therefore removed the sentence completely: Page 4, para 3.

The relationship of the SNP rs2734647 with the SNP (c.*3638A>G) should be explained for non-genetic experts.

c.*3638A>G is the nomenclature of the SNP rs2734647 position in the gene. This has now been clarified in the text, page 9, para 1.

Figures: Fig 4: The schematic representations of Mecp2 isoforms is confusing. These are splicing isoforms and the existence of them is dependent on intron elimination; thus depiction of introns is incorrect. The genomic organization of the locus should be presented with the location of the considered SNPS.

This has now been done. See new Figure5A.

Fig 6A: the meaning of the numbers surrounding the miRNA sequence should be clarified.

This has now been added to the Figure6 legend.

Fig 6B and C: note which baseline was used for statistical significance determinations (negative control or the alternative allele?)

This information has now been added to the Figure6 legend.

Referee #3 (Comments on Novelty/Model System):

The authors present human data indicating an association between decreased MECP2 expression, SNP, and clinical features, whereas they present animal data showing mild overexpression of MECP2 leading to a phenotype. They do not discuss this difference adequately.

This is unfortunately a total misunderstanding. The human data revealed increased aggression in case of non-regulability of gene expression (rs 2734647 - C carriers: no miR-511-mediated *MECP2* downregulation), and therefore increased expression. This is consistent with the effect in FVB mice, also being more aggressive in case of overexpression. See sketch below.

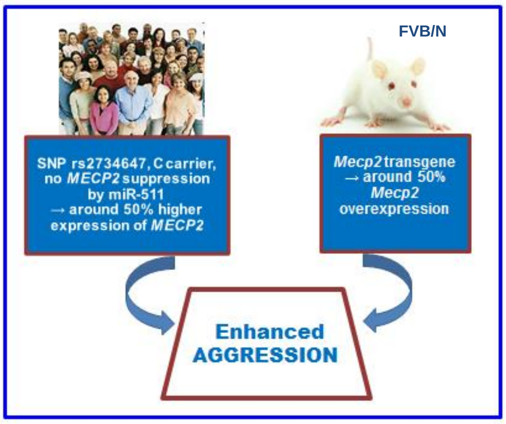

This has now been made clearer in the manuscript: Abstract, impact section, discussion.

Referee #3 (Remarks):

The authors provide additional characterization of a mouse with a 50% overexpression of MeCP2 protein. They identify an increased susceptibility to seizures, decreased locomotor activity, strain dependent modification of aggressive behavior, disrupted social behaviors, and enhanced acoustic startle response. The authors also identify polymorphisms in MECP2 that correlate with increased surrogate measures of aggressive behavior in human male schizophrenia patients. Additionally the authors propose and test a hypothesis regarding microRNA regulation of MECP2 as the mechanism contributing to the altered aggression measures. A relationship between MeCP2 function and aggressive behavior, social interactions, and motor function have been demonstrated in literature cited by the authors. Furthermore, 50% changes in MeCP2 expression have been demonstrated to cause behavioral abnormalities in mice in a 2008 HMG paper by Samaco et al (which the authors should cite) and in the Kerr paper already cited by the authors.

We have now included the respective paper of Samaco et al which, however, deals with a 50% expression REDUCTION! Page 3, para 2 and page 3, para 3.

The major novelty of the paper is in the identification of MECP2 polymorphisms that modify aggressive behavior in humans, and the identification of the interaction between miR-511 and the 3'UTR of MECP2 as the effector of this phenotype.
The human findings merit publication, however several concerns regarding methods and interpretations of the data should be addressed.

We thank the reviewer for his encouraging remarks.

Concerns:
1. The authors modified the protocol used to test aggressive behavior in the C57 mouse strain by placing the mice in a warmer environment before and during the test. However, no specific precedent is given/cited for why those changes would be expected to increase the aggressive behavior to detectable levels in the mice. Citation or empirical evidence should be added.

As the reviewer will see, we had performed the aggression testing at first in similar conditions in both strains (see new Table1). We did this test even in different age groups. However, when the protocol used for testing aggression in male FVB/N was applied to male C57BL6/N, not the slightest attack was registered over a 5min observation period (Table 1b). This result was not totally surprising, since male C57BL6/N mice are generally less aggressive compared to male FVB/N (Mineur & Crusio, 2002; Pugh et al, 2004).

The ambient temperature is known to influence aggression in mice (Greenberg, 1972; Gaskill et al., 2012). Therefore, we modified the protocol for the male C57BL6/N by introducing a warmer temperature (a warming pad set to 38°C) and prolonged the observation period to 10min. This finally gave the reported aggression results. This has now all been made clearer in the paper, page 5 para 3; page 6, para 1.

2. Control social behavior tests involving follow/chase, sniffing, nest building etc. are only presented for the C57 strain and are used to provide support for the difference in aggressive behavior between the strains. However, the diminished activity in the C57 strain could also be an explanation.

As presented in the new large overview Table1, there is no difference in activity between genotypes in the open field which is a more adequate readout to compare with activity in the resident-intruder paradigm. LABORAS measures spontaneous home cage behavior overnight. This is why it is unlikely that diminished activity played any role here.

Similar social behavior tests should be presented for the FVB strain for a more effective argument regarding strain (genetic background) effects on social behavior.

As also presented in Figure2 and the new Table1a, the attack latency of the resident male mice of the FVB/N background was considerably shorter compared to C57BL6/N mice, making such detailed observation basically impossible.

3. The inclusion of the female mouse data is appreciated. However, because the transgene is not confirmed to be localized to the X chromosome, translational relevance is diminished by lacking the ability to model mosaicism from XCI.

We feel that it was still important to compare genders since the transgene increases the gene dose but should not necessarily affect the expression of the X-chromosomal endogenous gene. But in appreciation of the reviewer's comment, we have now added this point as a limitation to the paper, page 15, para 1, lines 10-12.

4. The language should be tempered when using the mouse results for rationale of the human investigation. The stated mouse data indicates increased MeCP2 expression may increase or decrease aggressive behavior in a strain dependent manner, but the authors insist on only using the increased aggression from the FVB strain as a justification to look for increased aggressive behavior in people. Humans are not an isogenic population, the authors fail to address the prediction of a population with reduced aggression suggested by the C57 mouse strain data (i.e. a bimodal aggression distribution when combining the different backgrounds).

We thank the reviewer for this very valuable comment. He is totally right. We have now added this comment to the paper, page 11, para 3.

5. Although the authors choice of the PANSS to indirectly assess aggressive behavior is supported in the literature, their selection of only 2 individual subscores appears excessively reductive. Empirical evidence or citation of these specific subscores as predictive of aggressive behavior would be advised.

We thank the reviewer again for his important remark. In fact, Cheung et al (*The Australian and New Zealand Journal of Psychiatry, 1997; 31:62)* compared aggressive and non-aggressive schizophrenia patients (aggression assessed by the Staff Observation Aggression Scale) with respect to single items of the Positive and Negative Syndrome Scale (see Table2 in the Cheung paper). The largest group difference was found for the PANSS item 'poor impulse control'. Strikingly, the aggressive group had an average score of 4 (range: 1-7) on this item. Even after controlling for the total level of psychopathology, the associations of 'poor impulse control' and aggressive behavior remained significant. Additionally, in a more recent prospective study, 'poor impulse control' measured by PANSS was found to be highly predictive of aggressive behavior (assessed by the Overt Aggression Scale) (Nolan et al, *Journal of Psychiatric Research. 2005; 39:109-15).* This has now also been added to the manuscript, page 20, para 2.

6. The language in the abstract and Impact sections should be tempered regarding interactions between MECP2 expression and genetic background. While the mouse data provides evidence of an interaction between genetic background and MeCP2 expression on behavior. The human data only provides evidence of a means by which genetic background can effect MeCP2 expression and result in behavior change.

We thank again for a very constructive comment which we have integrated in abstract (page 2) and impact section, page 25, para 3.

7. The authors should be aware that the rs2734647 variant has been associated with increased risk of schizophrenia in a Han Chinese population (Wong et al. Schizophr Bull. 2013), since they claim it does not increase risk in the European Caucasion population used in their study.

Whereas our own data does not suggest a role of rs2734647 in the etiology of schizophrenia, we thank the reviewer for making us aware of the brand-new study by Wong et al, reporting a suggestive association of the SNP with schizophrenia in a Han Chinese population, even if the association does not reach genome-wide significance and requires further replication. We added this information to the manuscript, considering power issues and possible population-specific associations. See page 12, para 2.

8. Given that the ancestral and predominant allele at the locus of rs2734647 is C and the T allele shows reduced expression, the hypomorph Mecp2tm1Bird mouse (Kerr 2008, Samaco 2008) may be a better model of the human MECP2 expression difference observed in this paper. This is one of the biggest problems with this paper-the animal work is based on mild overexpression whereas the human condition mentioned is based on mild decreased expression. The authors cannot claim a direct relationship between these two-at best they can make the claim that EITHER mild over or under expression can lead to a phenotype, either in mice (under and over) or people (only evidence in people).

This is again an unfortunate misunderstanding. While the T allele shows reduced expression, it is the C allele that is associated with increased aggression in the human sample. Thus, consistent with the FVB mouse data, increased relative expression is associated with increased aggression. As this is suggested to be one of the biggest problems with the paper, it is important to point out that this criticism is based on a misunderstanding. See sketch above. Moreover, we have now tried to make this clearer in the manuscript: See abstract, impact section and discussion.

2nd Editorial Decision 18 January 2014

Thank you for the submission of your revised manuscript to EMBO Molecular Medicine. We have now received the enclosed reports from the referees that were asked to re-assess it.

As you will see, while referees 1 and 3 are now supportive of publication, referee 2 remains opposed to it. It appears that despite their opposite recommendations, both referees 1 and 2 are similarly concerned by the limited supportive nature of the mouse model for the human data. Nevertheless, as the findings remain of interest, we would like to suggest rewriting the manuscript in part in order to separate both sets of data (human and mouse) without pushing a link between the two, but highlighting respective results on their own, as implied by referee 1.

Would you be willing to modify your manuscript accordingly, we will be happy to move forward with the study publication. However, for now, we do find referee 2's points valid and using the mouse data as a model for the human findings is not fully justified by the findings.

***** Reviewer's comments *****

Referee #1 (Remarks):

The revised manuscript is improved. The data are interesting because of correlations in humans between variant and phenotype, and the phenotype in mice. Although the animal data are not exactly consistent with human data the value of the paper lies in showing that 50% increase in MeCP2 has an effect.

Referee #2 (Remarks):

The main issue in this paper remains the merger of somewhat disparate data. They demonstrate that the mild over expression of Mecp2 in mice has some mild phenotypic effects in behavior (aggression), which has some strain dependence, and then that a SNP in the 3'UTR which leads to decreased Mecp2 results in decreased aggression. The authors keep trying to flip the interpretation of the human data to match the mouse data, trying to imply that increased aggression is caused by the common SNP, rather than the reality which is the uncommon SNP leads to decreased MeCP2 protein and decreased aggression. The baseline phenotype cannot be in the rare SNP, it should be in the common SNP. In other words, they have nice evidence to show that there are non-coding mutations in the MECP2 locus that lead to decreased Mecp2 expression and behavior changes.

For the logic that the authors are trying to use, they would need to identify a rare SNP that leads to increased expression of Mecp2 and is associated with increased aggression.

One big issue is the meaning of the clinical phenotyping of aggression. I suspect that the cases associated with the common SNP (and thus "normal" MeCP2 levels) have what would be considered "normal" aggressive behavior, whereas the uncommon SNP with lower MeCP2 expression have what appears to be below normal levels of aggression.

Finally, aside from some strain differences, the behavior abnormalities they report in the mice are not novel, and have been reported by the group previously.

Referee #3 (Remarks):

The study is interesting in that associates aggressive social behavior with MECP2 genotype, genetic background and expression changes.

Some of the issues raised in the critique were responded satisfactorily.

Unfortunately, in the animal model, the fact that the two mouse strains (FVB and C57) are not polymorphic for the Mecp2 3'UTR allele or the miR-511 sequence does not add to their hypothesis. They sort of selectively choose one of them to support their idea of increased expression-increased aggression. If their transgenic model was of underexpression they might have commented on a decreased expression-decreased aggression?

The human data is still interesting.

2nd Revision - authors' response 23 January 2014

Thank you very much for having our manuscript "Mild expression differences of MECP2 influencing aggressive social behavior" again reviewed by the expert referees of EMBO Molecular Medicine.

Following your suggestion, we have now partly modified our paper in order to even more separate both sets of data (human and mouse). All changes are highlighted in yellow in the re-revised manuscript.

We feel that we achieved what you asked for, i.e. not pushing a link between the two data sets, but highlighting respective results on their own.

We hope that the re-revised version of the paper is now suitable for publication in EMBO Molecular Medicine.

We are looking forward to your response.
